# Attenuation of Cardiac Dysfunction in the Collagen-Induced Arthritis Mouse Model Mediated by a Reactive Aldehyde Species Inhibitor

**DOI:** 10.3390/ijms27198478

**Published:** 2026-09-23

**Authors:** Wenxian Zhou, Hannah J. Johnson, Michael J. Duryee, Ali Namvaran, Aradhana Thapa, Cole Blasing, Amy Nelson, Engle E. Sharp, Carlos D. Hunter, Bryan Hackfort, Bryant R. England, Tate M. Johnson, Daniel R. Anderson, Adam Lazorchak, Robert G. Bennett, Jill A. Poole, Keshore Bidasee, Geoffrey M. Thiele, Ted R. Mikuls

**Affiliations:** 1 Department of Internal Medicine, Division of Rheumatology & Immunology, University of Nebraska Medical Center, Omaha, NE 68198, USA; wezhou@unmc.edu (W.Z.); hannjohnson@unmc.edu (H.J.J.); mduryee@unmc.edu (M.J.D.); arthapa@unmc.edu (A.T.); cblasing@unmc.edu (C.B.); esharp@unmc.edu (E.E.S.); cdhunter@unmc.edu (C.D.H.); bryant.england@unmc.edu (B.R.E.); tate.johnson@unmc.edu (T.M.J.); gthiele@unmc.edu (G.M.T.); 2 Veterans Affairs Nebraska-Western Iowa Health Care System, Omaha, NE 68105, USA; rgbennet@unmc.edu; 3 Department of Pharmacology & Experimental Neuroscience, University of Nebraska Medical Center, Omaha, NE 68198, USA; ali.namvaranabbasabad@cuanschutz.edu (A.N.); kbidasee@unmc.edu (K.B.); 4 Department of Internal Medicine, Division of Allergy and Immunology, University of Nebraska Medical Center, Omaha, NE 68198, USA; amyjnelson@unmc.edu (A.N.); japoole@unmc.edu (J.A.P.); 5 Department of Cellular and Integrative Physiology, University of Nebraska Medical Center, Omaha, NE 68198, USA; bryan.hackfort@unmc.edu; 6 Department of Internal Medicine, Division of Cardiovascular Medicine, University of Nebraska Medical Center, Omaha, NE 68198, USA; danderso@unmc.edu; 7 Aldeyra Therapeutics, Lexington, MA 02421, USA; alazorchak@aldeyra.com; 8 Department of Internal Medicine, Division of Diabetes, Endocrinology and Metabolism, University of Nebraska Medical Center, Omaha, NE 68198, USA; 9 Department of Biochemistry and Molecular Biology, University of Nebraska Medical Center, Omaha, NE 68198, USA

**Keywords:** collagen-induced arthritis, rheumatoid arthritis, heart failure, cardiac dysfunction, reactive aldehyde species, malondialdehyde acetaldehyde

## Abstract

Individuals with rheumatoid arthritis (RA) experience increased risk of heart failure. Malondialdehyde–acetaldehyde (MAA) adducts are overexpressed and co-localize with citrullinated (CIT) proteins in RA myocardium. This study investigated whether reactive aldehyde species (RASP) inhibitors prevent MAA formation in vitro and attenuate cardiac dysfunction in a collagen-induced arthritis (CIA) mouse model. Among eight RASP inhibitors screened, ADX-246 demonstrated the greatest inhibition of MAA formation in vitro. Mice with early CIA (week 5) exhibited elevated levels of myocardial MAA adducts, myocardial inflammation, and reduced ejection fraction, but normal cardiac collagen deposition and diastolic function vs. control. By weeks 10–12, mice with chronic CIA demonstrated increased levels of myocardial MAA and CIT adducts, perivascular collagen deposition, and diastolic dysfunction. ADX-246 treatment reduced myocardial levels of MAA and CIT adducts, inflammation, fibrosis, and cardiac dysfunction in CIA. While ADX-246 reduced weight loss and attenuated arthritis severity, it did not reduce serum autoantibody levels or serum markers of inflammation or fibrosis. Indices of diastolic dysfunction significantly correlated with myocardial MAA and CIT levels, collagen deposition, arthritis severity, and weight loss. These findings support the hypothesis that targeting myocardial RASP and MAA formation may represent a novel therapeutic approach for preventing cardiac dysfunction in RA.

## 1. Introduction

Heart failure (HF) is one of the leading causes of morbidity and mortality among patients with rheumatoid arthritis (RA), a systemic autoimmune disease affecting approximately 0.5–1% of the adult population [1,2,3,4]. Multiple mechanisms have been proposed to drive this increased risk of cardiovascular disease including systemic inflammation, fibrosis, endothelial dysfunction, oxidative stress, and post-translational modifications (PTMs) [1,5,6]. However, despite advances in cardiovascular care and RA therapy, the prognosis of HF in RA remains suboptimal in comparison to non-RA populations. There are currently no therapies approved for the prevention or treatment of RA-related HF, underscoring the incomplete understanding of the precise molecular mechanisms driving RA-HF [7,8,9].

PTMs have been extensively implicated in RA pathogenesis through the promotion of autoantibody generation, local inflammation, and fibrosis. Citrullination (CIT), a PTM catalyzed by peptidyl arginine deiminase (PAD) enzymes, is one of the most well-characterized PTMs in RA, generating neoepitopes that are targeted by anti-citrullinated protein antibodies (ACPAs), which are highly specific for RA [10]. ACPA targets citrullinated forms of fibrinogen [11], vimentin [12], and type II collagen [13], among other proteins. Anti-cyclic citrullinated peptide (CCP) antibodies, a commercial measure of ACPA, have been associated with impaired cardiac diastolic and systolic function and increased left ventricular mass [14,15,16,17], suggesting a potential role in promoting cardiac remodeling that leads to HF. In addition, one animal study reported increased myocardial citrullination and reduced calcium sensitivity in the collagen-induced arthritis (CIA) mouse model of RA, implicating a role of citrullinated proteins in the development of cardiac dysfunction in this model [18]. Moreover, citrullinated proteins are significantly increased in myocardial tissue of RA patients compared to controls, further underscoring its potential role in promoting cardiovascular complications in RA [19,20].

Malondialdehyde acetaldehyde (MAA), a PTM structurally distinct from CIT, also contributes to RA-associated complications [21]. A byproduct of lipid peroxidation, MAA is generated via the spontaneous reaction of malondialdehyde and acetaldehyde on N-terminal amino groups or epsilon amino groups of lysine residues. The MAA epitope induces robust autoantibody and T cell responses in mice even in the absence of adjuvant [22]. Elevated serum concentrations of anti-MAA antibodies targeting modified fibrinogen, albumin, vimentin, and collagen have been reported in RA [23,24]. Notably, IgA anti-MAA antibodies have been shown to improve the 10-year prediction of coronary atherosclerosis in RA, beyond the prediction afforded by a standard cardiovascular risk calculation [25]. In addition to eliciting systemic autoimmunity, MAA adducts are enriched in the hearts of RA patients and co-localize with citrullinated proteins in regions of perivascular fibrosis [20]. MAA- and CIT-modified proteins have also been shown to synergistically activate macrophages to secrete soluble factors that promote inflammatory and fibrotic responses in human coronary artery endothelial cells [20]. Inhibition of citrullination via PAD inhibition has recently been shown to attenuate arthritis severity and prevent the onset of cardiac dysfunction in CIA mice [26]. However, whether inhibitors of MAA formation might also prevent cardiac dysfunction in CIA mice is unknown.

ADX compounds are novel small-molecule reactive aldehyde species inhibitors (RASPis) that bind to RASP (i.e., malondialdehyde and acetaldehyde) and prevent the formation of MAA adducts in cell culture systems and animal tissues [27,28,29,30]. Though MAA has been implicated in the pathogenesis of RA (and its cardiovascular complications), there have been no studies examining the potential efficacy of RASPis in the treatment of RA.

In this pre-clinical study, we tested the hypotheses that ADX treatment in mice with CIA would attenuate myocardial MAA adduct formation, reduce myocardial inflammation and fibrosis, and prevent the development of cardiac dysfunction. We also tested the hypotheses that ADX administration would be associated with reductions in systemic inflammation, autoantibody formation, weight loss, and arthritis severity.

## 2. Results

### 2.1. ADX-246 Prevented MAA Formation In Vitro

The effectiveness of eight ADX molecules in scavenging RASP and preventing MAA formation was assessed using an in vitro assay (Figure 1A). ADX-207, -246, -253, and -629 all significantly decreased MAA adduct formation at 0.5 mM concentration or above (Figure 1B; *p* < 0.05 vs. no ADX). The compounds ADX-248 and -743 decreased MAA formation only at concentrations of 1.0 mM or above (Figure 1B; *p* < 0.001 vs. no ADX). In contrast, ADX-203 and -210 failed to attenuate MAA formation at all concentrations tested (Figure 1B). Of the eight compounds tested, ADX-246 was the most potent in inhibiting MAA formation (Figure 1B; *p* < 0.001 vs. all other ADX at 1.0 mM concentration). Two millimolar of ADX-246 inhibited 97.3% ± 0.6% MAA formation in vitro (Figure 1B). ADX-246 was selected for subsequent animal experiments given its relative superiority in preventing MAA formation.

### 2.2. ADX-246 Attenuated Weight Loss and Decreased Arthritis Severity in Chronic CIA

All mouse groups had comparable weights at week 0 and week 5 (Figure 2A). However, CIA mice experienced significant weight loss between weeks 5 and 12 (Figure 2A; *p* < 0.01 CIA vs. control). Treatment with ADX-246 significantly attenuated this weight loss (Figure 2A; *p* < 0.05 CIA-ADX vs. CIA). No obvious off-target effect (such as premature death, visible distress, or gross abnormality in organs during dissection) was observed in mice treated with ADX-246.

CIA mice had earlier arthritis onset compared to CIA mice treated with ADX-246 (Figure 2B; onset at week 3 vs. week 6). However, ADX-246 did not prevent arthritis onset as mice from both the CIA and CIA + ADX groups had 100% arthritis incidence by week 8 (Figure 2B). While having comparable arthritis severity during the first 10 weeks, ADX-246 significantly reduced the arthritis severity score of CIA mice at weeks 11 and 12 (Figure 2C; *p* < 0.05 CIA-ADX vs. CIA).

### 2.3. ADX-246 Attenuated the Onset of Cardiac Dysfunction and Perivascular Fibrosis in CIA

Measurements of cardiac function at baseline, week 5, and week 10 are detailed in Table S1 and Figure S1. Five weeks after CIA induction (early CIA), CIA mice had no significant changes in indices of diastolic function (Figure 3). However, mice with early CIA demonstrated significant decreases in ejection fraction and fractional shortening (Figure 4; *p* < 0.05 CIA vs. control), suggestive of systolic dysfunction. Treatment with ADX-246 prevented the systolic dysfunction in the early CIA mice (Figure 4; *p* < 0.05 CIA-ADX vs. CIA).

At week 10 (chronic CIA), mice with CIA had recovered systolic function (Figure 4). In contrast to early CIA, mice with chronic CIA demonstrated decreases in E:A ratio and LVEDD, increased IVRT, and decreased cardiac output (Figure 3; *p* < 0.05 CIA vs. control). Collectively, these results indicated that hearts from CIA mice were stiffer and exhibited impaired relaxation, suggestive of grade 1 diastolic dysfunction [31,32]. Notably, treatment with ADX-246 normalized the E:A ratio, LVEDD, IVRT, and cardiac output (Figure 3; *p* > 0.5 CIA-ADX vs. control).

Because cardiac fibrosis is a major contributor to diastolic dysfunction, Masson’s trichrome stain was performed to quantify the collagen deposition in the hearts of all mice. Early CIA mice showed no significant changes in perivascular or interstitial collagen deposition in left ventricular tissues compared to that of control mice at week 5 (Figure 5A). In contrast, chronic CIA mice had no significant changes in interstitial collagen deposition but demonstrated a significant increase in perivascular collagen deposition at week 12 (Figure 5B; *p* < 0.05 CIA vs. control). Immunohistochemistry further demonstrated that both collagen I and collagen III were elevated in the perivascular regions of chronic CIA mice (Figure 6; *p* < 0.001 CIA vs. control). Treatment with ADX-246 prevented increased perivascular collagen deposition in the chronic CIA mice (Figure 5B and Figure 6; *p* < 0.01 CIA-ADX vs. CIA).

### 2.4. ADX-246 Attenuated the Myocardial MAA and CIT Deposition in CIA

MAA and CIT adducts were quantified in heart tissues because of their role as autoantigens and promotors of local inflammation and fibrosis in RA [20,21]. At week 5, CIA mice demonstrated increased myocardial expression of MAA adducts compared to control (Figure 7A; *p* < 0.0001). In contrast, myocardial CIT deposition was not significantly different between groups (Figure 7A). At week 12, CIA mice demonstrated increased myocardial expression of both MAA and CIT adducts compared to control (Figure 7B; *p* < 0.0001) with evidence of moderate co-localization (r^2^ = 0.64). Treatment with ADX-246 significantly attenuated the level of MAA adduct expression in the myocardium of CIA mice at week 5 and decreased levels of both MAA and CIT expression in the myocardium at week 12 (Figure 7; *p* < 0.0001 CIA-ADX vs. CIA).

### 2.5. ADX-246 Prevented Myocardial Inflammation in Early CIA

Gene expression of *Tnfα*, *Il1β*, *Il10*, *Il6*, *Tgfβ1*, *enos*, *Acta2*, and *Col1a1* are examined because of their established roles in promoting inflammation and fibrosis of myocardial tissues [33,34,35]. At week 5, mice with CIA demonstrated significant increases in the gene expression of *Tnfα*, *Il1β*, and *Il10* in left ventricular tissue (Figure 8A; *p* < 0.01 CIA vs. control). Gene expression of *Il6*, *Tgfβ1*, *enos*, *Acta2*, and *Col1a1* was not significantly changed in the early CIA compared to control (Figure 8A). Treatment with ADX-246 decreased the gene expression of *Tnf* (*p* < 0.05), *Il1β* (*p* = 0.13), and *Il10* (*p* < 0.001) in the myocardium of early CIA mice (Figure 8A), though the treatment-related difference in *Il1β* expression did not achieve statistical significance. At week 12, gene expression of *Tnfα*, *Il1β*, and *Il10* returned to baseline in the CIA group, and there were no significant differences in myocardial gene expression of *Tnfα*, *Il1β*, *Il10*, *Il6*, *Tgfβ1*, *enos*, *Acta2*, and *Col1a1* across the four groups (Figure 8B).

### 2.6. ADX-246 Did Not Impact Serum Autoantibodies or Measures of Inflammation or Fibrosis in CIA

At week 5 and week 12, CIA mice demonstrated elevations in circulating TNF-α and anti-CCP antibody compared to control (Figure 9; *p* < 0.05 CIA vs. control). CIA mice had a non-significant increase in anti-HSA-MAA antibody level at week 5 (Figure 9A; *p* = 0.14 CIA vs. control) that returned to baseline at week 12. In addition, CIA mice had significantly increased anti-HSA-CIT antibody levels at week 12 (Figure 9B; *p* < 0.01 CIA vs. control). Treatment with ADX-246 failed to meaningfully attenuate any of these serum markers in CIA compared to sham treatment (Figure 9). In addition, CIA mice receiving ADX-246 had significantly increased serum levels of IL-6, pentraxin, and TGFβ1 compared to control at week 12 (Figure 9B; *p* < 0.05).

### 2.7. Indices of Diastolic Dysfunction Correlate with Arthritis Severity, Weight Loss, Perivascular Fibrosis, and Interstitial Fibrosis in Chronic CIA

Arthritis score, weight loss, perivascular fibrosis, interstitial fibrosis, and levels of myocardial MAA and CIT deposition at week 12 were significantly correlated with worsened indices of diastolic dysfunction measured at week 10. Correlations were most striking for the E:A ratio (Figure 10). This included a strong correlation between the E:A ratio and myocardial CIT antigen levels (r = −0.75, *p* < 0.001). In addition, there was evidence of moderate-to-strong correlations between the E:A ratio and arthritis score (r = −0.69, *p* < 0.001), myocardial MAA antigen levels (r = −0.66, *p* = 0.001), change in body weight (r = 0.64, *p* = 0.002), and interstitial fibrosis (r = −0.55, *p* = 0.010) as well as a moderate correlation with perivascular fibrosis (r = −0.48, *p* = 0.027). Similar correlation patterns were found with other indices of diastolic dysfunction, such as IVRT and LVEDD (Table S2).

## 3. Discussion

Although our recent study revealed increased levels of MAA-modified proteins in cardiac tissues from patients with RA and HF [20], whether MAA-modified proteins contribute to HF in RA remains unknown. In the present study, we investigated whether the novel RASPi, ADX-246, inhibits MAA formation and attenuates cardiac dysfunction in a mouse model of RA. We have demonstrated that treatment of CIA with ADX-246 attenuated chronic arthritis severity in addition to myocardial formation of MAA-modified proteins, cardiac inflammation, perivascular fibrosis, and ultimately cardiac dysfunction. Consistent with previous reports [26,36,37,38,39], mice with CIA exhibited early transient reductions in ejection fraction at week 5, followed by a significant decrease in diastolic function during the more chronic stages of disease. The development of diastolic dysfunction in CIA closely resembles the clinical phenotype observed in RA populations, wherein the risk of developing heart failure with preserved ejection fraction (HFpEF) is disproportionate to the risk of heart failure with reduced ejection fraction (HFrEF) [7,40,41]. Moreover, we demonstrated for the first time that myocardial accumulation of MAA-modified proteins and cardiac inflammation occur during early CIA, preceding increases in myocardial formation of citrullinated proteins, cardiac fibrosis, and diastolic dysfunction. Importantly, pharmacologic inhibition of RASP with ADX-246 attenuated myocardial formation of MAA-modified proteins, inflammation, and systolic dysfunction in mice with early CIA, and it subsequently prevented cardiac fibrosis and diastolic dysfunction in mice with chronic CIA.

MAA-modified proteins are generated via a non-enzymatic reaction of malondialdehyde and acetaldehyde with protonated amino groups on proteins [21]. In addition to promoting autoantibody production and local inflammation, MAA-modified proteins have recently been shown to upregulate PAD enzymes in macrophages, thereby increasing the formation of citrullinated proteins [42]. In this study, ADX-246 reduced myocardial accumulation of both MAA-modified and citrullinated proteins, suggesting that inhibition of MAA formation interrupted downstream pathways leading to citrullination. Both MAA-modified and citrullinated proteins were shown to be overexpressed in perivascular regions of RA myocardium and strongly co-localize with each other, suggesting that they may modify similar proteins in the myocardium of RA patients [20]. In previous work, it has been shown that proteins modified by both MAA and CIT activate macrophages and induce inflammatory and fibrotic responses in human coronary artery endothelial cells, potentially contributing to the increased perivascular fibrosis in the myocardium of RA patients and CIA mice [20]. Collectively, the current findings suggest that the cardioprotective effects of ADX-246 are likely mediated through scavenging RASPs in heart tissue, thereby preventing the formation of MAA-modified and citrullinated proteins, reducing myocardial inflammation, and limiting perivascular fibrosis. Taken together with prior in vitro findings, these data support a pathogenic role of RASP and MAA-modified proteins in the underlying mechanisms of cardiac dysfunction in RA. An intriguing finding of this study is that the cardioprotective effects of ADX-246 occurred despite minimal effects on autoantibody production or circulating inflammatory and profibrotic markers in CIA mice, further suggesting that its cardioprotective effects are primarily mediated through inhibition of local myocardial formation of MAA-modified proteins and inflammation rather than through suppression of systemic inflammation.

In studies of mice with CIA at five weeks, myocardial gene expression of *Tnfα*, *Il1β*, and *Il10* was significantly increased compared to controls, indicative of myocardial tissue inflammation early in the disease process. Similar increases in myocardial *TNFα* and *IL1β* have been reported in patients with inflammatory rheumatic diseases compared to non-inflammatory rheumatic disease controls, supporting the clinical relevance of this model [43]. In contrast, myocardial *Il6* expression was not elevated in our model, consistent with recent studies demonstrating limited cardioprotective effects of IL6 receptor blockade compared with TNF inhibition in rats with CIA [44,45,46]. The increased expression of *Il10* in CIA mice likely represents a compensatory anti-inflammatory response to myocardial injury [47,48]. In addition, the early increases in myocardial *Tnfα* and *Il1β* expression in CIA mice occurred concurrently with the early decrease in ejection fraction. Similarly, myocardial TNFα and IL1β levels are increased in patients with HFrEF [49], and these cytokines have been shown to promote contractile dysfunction in both cell culture and rodent models [50,51,52,53,54]. Together, these findings suggest that TNFα and IL1β are key mediators of local myocardial inflammation and cardiac dysfunction in RA.

The progression from early myocardial inflammation to perivascular fibrosis observed in this study is consistent with the current understanding of HFpEF pathogenesis [34,55]. While no significant increase in collagen deposition was detected during early CIA, mice with chronic disease developed marked perivascular fibrosis characterized by increased collagen I and collagen III deposition accompanied by impaired diastolic function. Collagen I and collagen III are the predominant extracellular matrix proteins produced by activated cardiac fibroblasts and myofibroblasts and are major contributors to myocardial stiffness and fibrosis in patients with HFpEF [55,56,57,58]. Interestingly, despite the increased collagen protein deposition observed in chronic CIA, myocardial expression of Tgfβ1, enos, Acta2, and Col1a1 was not significantly increased in our CIA model at either week 5 or week 12. This discrepancy likely reflects the dynamic nature of cardiac remodeling. Previous studies have reported increases in myocardial gene expression of Acta2, Col1a1, and Col3a1 at week 7 after CIA induction [38,39], suggesting that the peak expression of these profibrotic genes may have occurred between our two study time points. Additional study will be needed to better define the temporal relationship of gene expression and protein deposition using this model.

Although ADX-246 produced robust cardioprotective effects, its impact on arthritis was comparatively modest. ADX-246 delayed arthritis onset and reduced arthritis severity during the late stages of disease but did not prevent arthritis development or significantly alter arthritis severity during the first 10 weeks following CIA induction. These findings are similar to those reported following pharmacologic inhibition of citrullination, in which delayed arthritis progression was observed despite limited effects on arthritis incidence [26]. Treatment with ADX-246 also attenuated the weight loss observed during chronic CIA. Similar to patients with RA, weight loss in CIA mice is believed to result from systemic inflammation and directly corresponds to arthritis severity [59,60,61,62]. Notably, ADX-246 preserved body weight despite yielding little to no effect on circulating inflammatory mediators, suggesting that the improvement in weight loss is unlikely due to its effect on systemic inflammation. Instead, reduced arthritis severity and cardiac dysfunction from ADX-246 may have improved physical activity and food intake, thereby limiting muscle wasting and weight loss in CIA. Because physical activity, food intake, and other metabolic parameters were not evaluated in this study, the precise mechanisms by which ADX-246 prevents weight loss in CIA remain uncertain and warrant further investigation.

The correlation analyses further support the existence of a close relationship between myocardial PTMs, arthritis severity, cardiac fibrosis, and diastolic dysfunction. Myocardial MAA and CIT levels were among the strongest correlates of diastolic dysfunction. Likewise, arthritis severity, weight loss, and cardiac fibrosis were all significantly correlated with measures of impaired diastolic function. Higher disease activity in patients with RA has been associated with an increased incidence and severity of cardiovascular complications [9,63,64], suggesting that common pathogenic mechanisms may underlie both joint destruction and cardiac dysfunction. One such mechanism may involve the accumulation of MAA and CIT adducts. Both modifications have been demonstrated to be enriched in the joints, lungs, and myocardium of patients with RA in previous work, where they promote local inflammation and fibrosis [20,23,65]. The strong association between myocardial MAA and CIT antigen deposition and diastolic dysfunction further supports their potential contribution to the underlying pathogenesis of RA-HF.

This study has limitations. Although our findings suggest that the myocardial accumulation of MAA-modified and citrullinated proteins may contribute to cardiac dysfunction in CIA mice, the specific proteins that are MAA-modified or citrullinated in heart tissues of patients with RA remain unknown. Citrullinated proteins, including vimentin, fibrinogen, and histones have been identified in tissues from patients with cardiovascular disease [66]. In addition, mass spectrometry has identified shared peptides between malondialdehyde (MDA)-modified and citrullinated vimentin and actin [67]. Citrullinated and MAA-modified proteins such as vimentin and fibrinogen have been reported to activate macrophages, fibroblasts, and endothelial cells in vitro [20,68,69,70], suggesting their potential role in promoting myocardial inflammation and fibrosis. Further studies are needed to identify proteins being increasingly citrullinated and/or MAA-modified in myocardial tissues of patients with RA to provide additional mechanistic insight into the relationship between these PTMs and cardiac dysfunction in RA.

While sample size was estimated based on similar studies using the CIA mouse model [26,37,38], no formal power calculation was performed prior to the study. The relatively small numbers of animals for between-group comparisons at each time point may limit the power of this study. In addition, only two terminal time points were examined in this study. Additional time points will be needed to be studied in the future to better define the temporal relationship between early myocardial inflammation and subsequent cardiac fibrosis.

Although the CIA mouse model is one of the most robust and widely used animal models for investigating RA pathogenesis and RA-associated cardiac dysfunction [26,36,37,38,39], the diastolic dysfunction observed was mild (grade 1) in severity. Therefore, additional animal models will be required to determine whether RASP inhibition is effective in treating more advanced stages of RA-associated heart failure. Though ADX compounds have been previously examined in a phase III clinical trial for the treatment of dry eye disease [27], supporting the potential for human translation of these findings, questions remain regarding the possible off-target effects with systemic delivery as well as optimal dosing and timing of this agent. The dosing of ADX-246 used in these experiments was selected based on unpublished data demonstrating that this dose effectively attenuated inflammatory cytokine release following LPS challenge in mice. Whether this represents the optimal dose in the CIA model remains unknown and will require further investigation to more precisely define. Likewise, because treatment was initiated before the development of cardiac dysfunction, it is unclear whether ADX-246 can reverse established cardiac dysfunction after disease onset. Future studies should optimize dosing regimens and evaluate the therapeutic efficacy of delayed treatment initiation.

In conclusion, this study provides the first in vivo evidence that pharmacologic inhibition of RASPs prevents myocardial MAA accumulation, attenuates cardiac inflammation and perivascular fibrosis, and attenuates cardiac function in a CIA mouse model. These findings identify myocardial RASP-mediated MAA formation as an early pathogenic event preceding cardiac fibrosis and dysfunction, suggesting it may contribute to the disease progression of RA-associated HFpEF. Collectively, our results suggest that reactive aldehyde scavenging represents a promising therapeutic strategy for preventing HF in RA and establish a foundation for future mechanistic and translational studies aimed at targeting this novel pathway.

## 4. Materials and Methods

### 4.1. In Vitro Assay Assessing the Impact of ADX on MAA Formation

An in vitro assay was performed to screen the efficacy of eight ADX compounds (all provided in-kind by Aldeyra Therapeutics, Lexington, MA, USA) in preventing MAA formation. Eight compounds were tested and included: ADX-246, -203, -207, -210, -248, -253, -629, and -743. The chemical structure, synthesis, and properties of the ADX compounds are detailed in U.S. patent numbers 9,687,481 and 18,568,566. The chemical structure, lot number, purity, and associated patent for each of these compounds are listed in Table S3. Briefly, all ADX compounds had >98% purity and were stored under −20 °C protected from light. The compounds share a common mechanism of action involving a direct covalent reaction with free aldehydes via a two-step reaction involving Schiff base formation followed by a ring closure, resulting in stable and non-reactive drug–aldehyde adducts. The individual compounds differ in their core chemical structures which may influence compound solubility, tissue permeability, pharmacokinetics and relative aldehyde species reactivity.

The ADX compounds were dissolved separately in 20% Captisol (Aldeyra Therapeutics) to generate 25 mM stock solutions. Captisol was selected as the delivery system because it has been shown by Aldeyra Therapeutics to be a suitable vehicle for solubilizing ADX molecules, does not interfere with ADX reactivity, and is suitable for both in vitro and in vivo applications (US patent #12,064,516). For each compound, freshly made ADX stock solution was then mixed with 1 mg/mL of human serum albumin (HSA; Akron Biotechnology LLC, Boca Raton, FL, USA) as the protein substrate along with 2.0 mM malondialdehyde (Millipore Sigma, Burlington, MA, USA) and 1.0 mM acetaldehyde (Millipore Sigma) in 0.1 M phosphate buffer (pH 7.2) as previously described to achieve final ADX concentrations of 2 mM, 1 mM, 0.5 mM, 0.1 mM, or 0 mM [23]. The 1 mL of reaction mixture was then incubated at 37 °C for 2 days to allow MAA adduct formation. Following incubation, the MAA modifications were detected and quantified by measuring fluorescence generated by its dihydropyridine ring structure (excitation 398 nm, emission 460 nm) using the SpectraMax iD3 instrument (Molecular Devices, San Jose, CA, USA). Relative fluorescence intensity compared to control solution (MAA formation solution without ADX) was calculated.

### 4.2. CIA Model, ADX Treatment, and Arthritis Scoring

For these pre-clinical studies, the CIA model was used. In addition to representing the most frequently used model for RA, mice with CIA have been demonstrated to recapitulate myocardial dysfunction that may complicate its disease course [26,36,37,38,39]. Arthritis-prone male DBA/1J mice (10–11 weeks old) were purchased from Inotiv (Lafayette, IN, USA). Male mice were selected for their robust susceptibility to both arthritis and cardiac dysfunction compared to female mice [26,36,37,38,39]. Mice were acclimated following receipt for one week prior to experiments, group housed in an independent ventilation cage system and provided sterile water and autoclaved chow ad libitum.

At the start of the experiment (day 0), mice were randomly assigned to one of four experimental groups (Figure 11): CIA receiving daily ADX-246 treatment (CIA + ADX; n = 9), CIA receiving daily vehicle control treatment (CIA; n = 11), control (no CIA) receiving daily ADX-246 treatment (ADX; n = 9), and control (no CIA) receiving daily vehicle control treatment (control; n = 9). Sample size was estimated based on similar studies using the CIA mouse model [26,37,38].

CIA was induced by intradermal injection of 100 μL of an emulsion containing 2.5 mg/mL complete Freund’s adjuvant and 5 mg/mL chicken type II collagen (Chondrex, Woodinville, WA, USA) approximately 2 cm distal from the base of the tail on day 0. On day 21, mice received a booster intradermal injection consisting of chicken type II collagen emulsified in incomplete Freund’s adjuvant at a site proximal to the initial injection. Control animals received 100 μL sterile saline intradermally on both day 0 and day 21.

Beginning on day 0, mice received daily (5 consecutive days/week) intraperitoneal injections of either 100 mg/kg ADX-246 (Aldeyra Therapeutics) dissolved in 20% Captisol (Aldeyra Therapeutics) or 20% Captisol dissolved in saline alone as a vehicle control. ADX-246 was selected for the animal study because it showed the greatest inhibition of MAA formation in vitro. The 100 mg/kg ADX-246 dose was selected based on unpublished data showing rapid ADX-246 clearance and good tolerability at a dose of 400 mg/kg or below in mice. Since this is a long-term model of inflammation, a daily dose of ADX-246 for 5 consecutive days per week was given to sustain effective systemic drug levels. ADX-246 repeatedly dosed at 100 mg/kg QD dose is below the twice daily, repeat-dose no-observed-adverse-effect level (NOAEL) in rats of 500 mg/kg (personal communications with Aldeyra).

Mice were assessed daily for the development of arthritis using a semiquantitative mouse arthritis scoring system provided by Chondrex (https://www.chondrex.com/documents/Scoring-System.pdf, accessed on 4 August 2026). Each paw was assigned a score ranging from 0 (no detectable inflammation) to 4 (severe swelling involving the ankle, foot, and digits). Scores from all four paws were averaged to generate a final arthritis severity score.

This animal study was approved by the University of Nebraska Medical Center Institutional Animal Care and Use Committee (IACUC# 25-052-10-FC) prior to experimentation. All experiments adhered to guidance from the U.S. Public Health Service Policy for the Humane Care and Use of Laboratory Animals; the USDA and the Animal Welfare Act; U.S. Government Principles for the Utilization and Care of Vertebrate Animals Used in Testing, Research and Training; the National Research Council of the National Academies Guide for the Care and Use of Laboratory Animals; and all other relevant local guidelines and policies. All mice included in this study remained in good health throughout the duration of the experiments, displaying normal body condition and behavior. None of the animals were excluded from data acquisition or subsequent analysis.

### 4.3. Echocardiography

Prior to the echocardiography, mice were anesthetized with 1–3% isoflurane (Cardinal Health, Dublin, OH, USA) and taped in the supine position on a heated 37 °C pad with feet leads. Anesthesia was maintained throughout the procedure via a nose cone. Transthoracic echocardiography (including generation of pulsed-wave Doppler, M-mode, and tissue Doppler images acquired and digitally stored in cine loops) was performed at week 0 (1 day prior to CIA induction), week 5 (early CIA), and week 10 (chronic CIA) to examine the time-course of cardiac function. Echocardiography was conducted using a Fujifilm Visual Sonics Vevo 3100 system (FUJIFILM VisualSonics, Toronto, ON, Canada), employing a MX550D transducer (Wohl Institute, London, UK) with a center frequency of 40 MHz and an axial resolution of 40 μm. The offline Program Vevo LAB 5.9.0 was then used to assess peak early and late diastolic transmitral velocities (E and A waves), E:E’ ratio, isovolumic relaxation time (IVRT), and left ventricular end diastolic diameter (LVEDD) as indices of diastolic function. Ejection fraction (EF), fractional shortening (FS), stroke volume, cardiac output, isovolumic contraction time (IVCT), and left ventricular end systolic diameter (LVESD) were assessed as indices of systolic function. Echocardiography measurements were analyzed by study personnel blinded to group assignment.

### 4.4. Animal Sacrifice and Tissue Collection

Mice were sacrificed at week 5, and the remaining mice were sacrificed at week 12. Under deep anesthesia, whole blood was collected from the axillary artery, and residual blood was removed from the heart tissue by perfusing the circulation with saline through the left ventricular apex. The heart was then excised and sectioned along the coronal plane. The ventral half of the heart was fixed in 4% paraformaldehyde for 24 h, dehydrated in 70% ethanol for 24 h, embedded in paraffin, sectioned into 4–5 μm slices and placed on glass slides. The dorsal half of the ventricular tissue was preserved in Allprotect tissue reagent (Qiagen, Hilden, Germany) for subsequent RNA extraction.

### 4.5. Histopathology and Immunohistochemistry of Myocardial Tissues

Paraffin-embedded heart tissues underwent paraffin removal with xylene for 5 min, followed by sequential rehydration in 100% ethanol, 95% ethanol, 80% ethanol, 50% ethanol, deionized water, and PBST for 1 min each. One section per donor was stained with Masson’s trichrome to detect collagen deposition indicative of tissue fibrosis. Another section from each donor underwent immunohistochemistry to stain for MAA, CIT, and collagen deposition. Sections were subjected to antigen retrieval using 10 mM citrate buffer (pH 6.0) for 40 min using a steamer. After blocking with 2% goat serum, sections were incubated overnight at 4 °C with primary antibodies (1:100 dilution in 2% goat serum). Primary antibodies included Zenon AF 594-labeled (Invitrogen, Carlsbad, CA, USA) rabbit polyclonal anti-MAA IgG antibody, mouse monoclonal anti-peptidyl-CIT IgM antibody (Millipore Sigma), rabbit Cy5 conjugated polyclonal anti-collagen I antibody (Bioss Inc., Woburn, MA, USA), and Zenon AF594-labeled rabbit polyclonal anti-collagen III antibody (Invitrogen). Collagen I and III were stained because they are the predominant extracellular matrix proteins produced by activated cardiac fibroblasts and myofibroblasts and are major contributors to myocardial stiffness and fibrosis in patients with heart failure [55,56,57,58]. Detection of anti-CIT antibody was carried out using AF488 AffiniPure Donkey anti-mouse IgM (1:1000; Jackson ImmunoResearch, West Grove, PA, USA). After a 45 min incubation with secondary antibody at room temperature, 4′,6-diamidino-2-phenylindole (DAPI) was applied, and samples were mounted with Fluoromount-G (Southern Biotech, Birmingham, AL, USA). Imaging was performed on an Echo Revolve Microscope (Discover Echo, San Diego, CA, USA).

Immunohistochemistry quantification and co-localization analysis were conducted as previously described using ImageJ (v2.14.0/1.54f) and the FIJI Coloc2 plugin (NIH, Bethesda, MD, USA) [23,65]. Briefly, mean pixel densities of antigens were quantified by removing background fluorescence and then using the “measure” function. Pearson’s R values (with no threshold) were generated as measures of co-localization. Quantification of perivascular and interstitial fibrosis was undertaken as described in previous publications [20,71,72]. Briefly, three representative images were taken of tissue from each donor at 20× magnification from both perivascular regions (regions containing blood vessels) and interstitial regions (regions not containing blood vessels). Mean intensity of collagen deposition (blue) in each image was quantified using the “color deconvolution” and “measure” functions in FIJI.

### 4.6. RT-PCR of Myocardial Tissues

Ventricular tissues were removed from the Allprotect solution and washed. RNA was extracted per manufacturer’s protocol using RNeasy Mini Kit (Qiagen). RNA concentrations were quantified using the Nanodrop 2000c (Thermo Fisher, Waltham, MA, USA) and then converted to cDNA using a High-Capacity RNA-to-cDNA kit (Applied Biosystems, Waltham, MA, USA). To generate the gene amplification mixture, cDNA was mixed with TaqMan Gene Expression Master Mix (Midsci, Fenton, MO, USA) and TaqMan Gene Expression Assays (Thermo Fisher). The following TaqMan primer/probe sets were used in real-time polymerase chain reaction (RT-PCR) analysis: *Tnfα*, *Il1β*, *Il10*, *IL6*, *Tgfβ1*, *enos*, *Acta2*, and *Col1a1* (Thermo Fisher). These genes were selected based on each having an established role in inflammation and fibrosis of myocardial tissue [33,34,35]. Gene expression was normalized to *Gapdh*. Normalized gene expression from each group was compared to that of the control group and presented as a relative quantification (RQ).

### 4.7. Serum Autoantibody and Biomarker Detection

Serum was derived from whole blood collected at the time of euthanasia. Serum antibodies directed against citrullinated HSA (HSA-CIT) and MAA-adducted HSA (HSA-MAA) were determined by ELISA as previously described [23]. Detailed ELISA methods are provided in the Supplementary Materials.

Since serum antibodies to HSA-CIT were present, additional confirmation was completed using a modified version of the human CCP ELISA (The Gold Standard Diagnostics Anti-CCP ELISA, Thermo Fisher). In brief, anti-CCP antibody was quantified by incubating the mouse serum onto the plate as per manufacturer instructions and then using HRP Goat anti-mouse IgG (Jackson Immuno Research) as the detection antibody. Plate absorbance at 450 nm was detected using an Epoch plate reader (Agilent, Santa Clara, CA, USA) and analyzed using Gen5 Softwarev.2.07 (Agilent).

To measure inflammatory and fibrotic responses in serum, the murine acute phase reactant protein pentraxin-2 and transforming growth factor (TGF)-β1 were quantified by ELISA according to the manufacturer’s instructions (R&D Systems, Minneapolis, MN, USA). In addition, mouse tumor necrosis factor (TNF)α, interleukin (IL)6, IL1β, and IL10 were quantified by U-Plex Custom Mouse Biomarker Assay according to the manufacturer’s instructions (Meso Scale Discovery, Rockville, MD, USA). The manufacturer and catalog number of each ELISA kit and the U-Plex system are listed in the Major Resources Table S4 in the Supplementary Materials.

### 4.8. Statistical Analyses

Comparisons between groups were analyzed using one-way ANOVA with Tukey’s post hoc test (95% confidence interval). To investigate factors associated with measurements of diastolic function, nonparametric Spearman correlation coefficients were generated using cross-sectional endpoint data pooled from all four groups of mice. Strengths of correlation were interpreted as moderate (r = 0.30–0.50), moderate-to-strong (r = 0.51–0.70) or strong (r > 0.70). Statistical significance was defined as *p* < 0.05 after multiple comparison adjustments. Graphs display mean ± standard deviation. Analyses were performed using GraphPad Prism 10.5.0 (San Diego, CA, USA).

### 4.9. Data Availability

The data supporting the findings of this study are available from the corresponding author upon reasonable request. All essential research materials listed in this method section are included in the Major Resources Table S4.

### 4.10. Involvement of Study Sponsors

ADX compounds and guidance on dosing were provided in-kind (Aldeyra Therapeutics). Study sponsors otherwise played no role in experimental design, data collection, analysis or data interpretation.

## Figures and Tables

**Figure 1:**
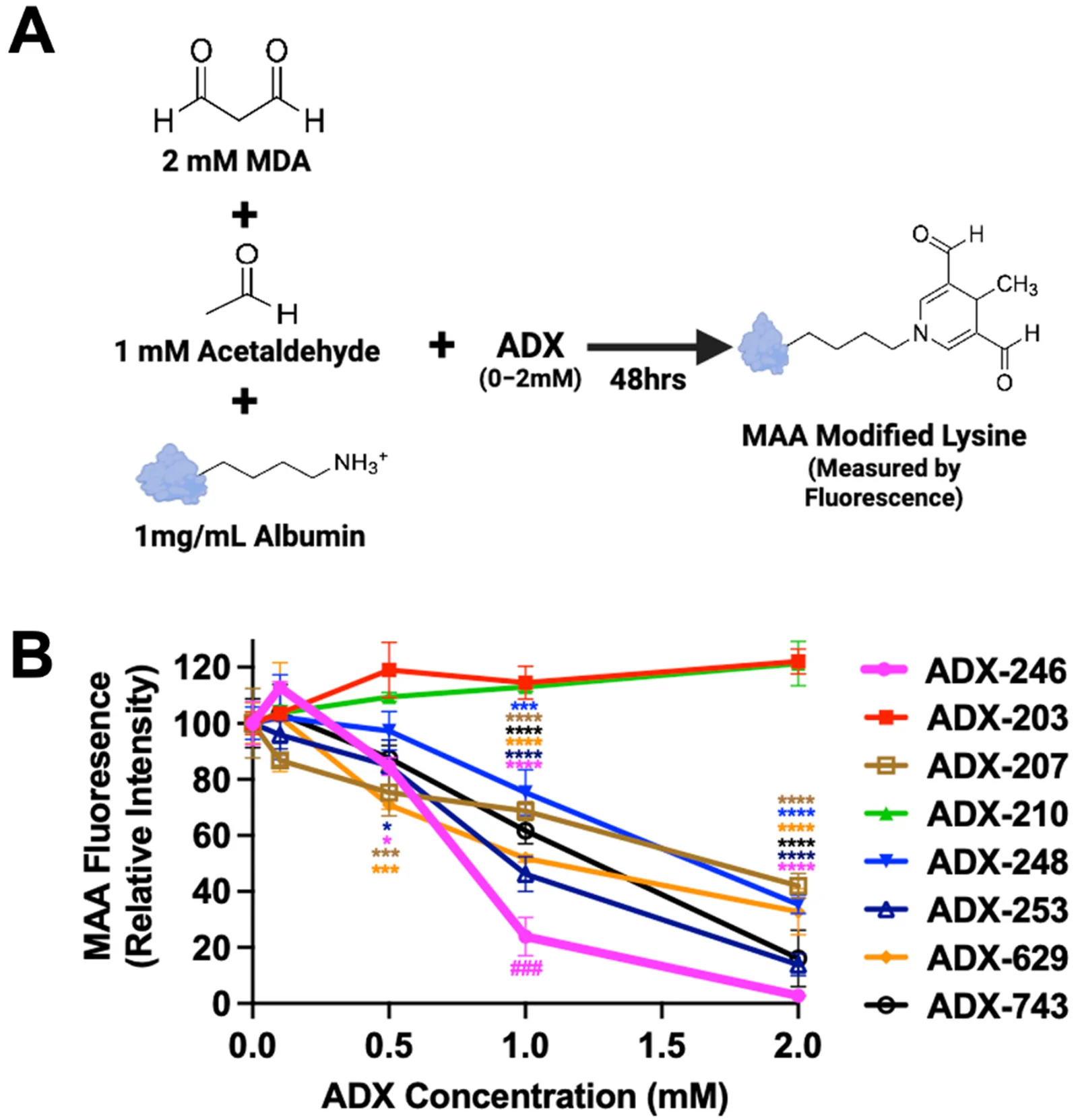
Effect of ADX Formulations on MAA Adduct Generation in vitro. (**A**) Study schema. (**B**) Levels of MAA formation following mixing MAA precursors with increasing concentrations of ADX are shown. The concentration of each compound shown in panel A represents its final concentration in the reaction mixture. Data shown on graphs are mean ± standard deviation. The error bar for ADX-246 at 2 mM concentration is not readily visible because the standard deviation at this concentration is 0.6%. For each ADX compound, difference in MAA formation between each ADX concentration and 0 mM ADX baseline are shown: * *p* < 0.05; *** *p* < 0.001; **** *p* < 0.0001. At each ADX concentration, difference in MAA formation between ADX-246 and all other ADX compounds are shown: ^###^ *p* < 0.001.

**Figure 2:**
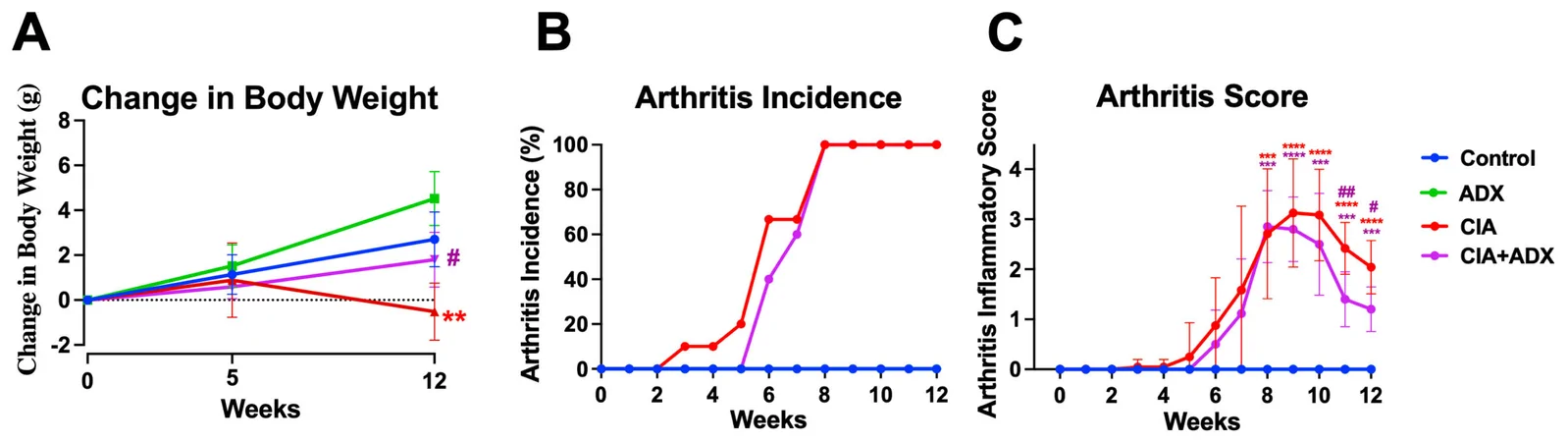
ADX-246 Attenuates Weight Loss and Arthritis Severity in Mice with Chronic CIA. (**A**) Change in body weight from baseline, (**B**) arthritis incidence, and (**C**) arthritis score are shown for control (n = 5), ADX (n = 5), CIA (n = 6), and CIA + ADX (n = 5). Differences between each treatment group vs. saline control are shown: ** *p* < 0.01, *** *p* < 0.001; **** *p* < 0.0001. Differences between CIA + ADX and CIA group are shown: # *p* < 0.05, ## *p* < 0.01. Data shown on graphs are mean ± standard deviation. No mice in control or ADX group developed evidence of arthritis over 12 weeks.

**Figure 3:**
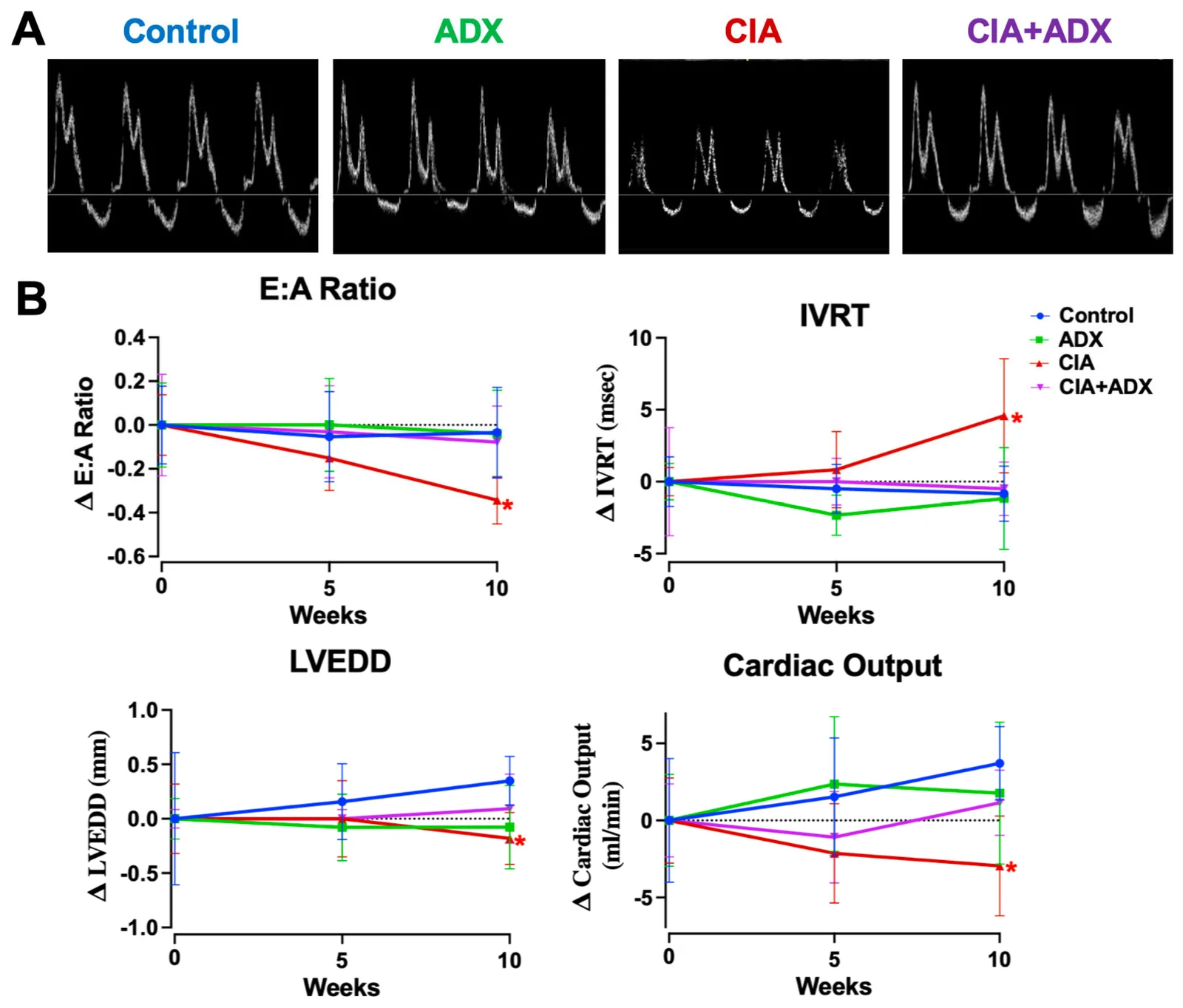
ADX-246 Attenuates Diastolic Dysfunction in Mice with Chronic CIA. (**A**) Representative pulsed-wave echocardiograms from control (n = 5), ADX (n = 5), CIA (n = 6), and CIA + ADX (n = 5) mice at week 10. (**B**) Longitudinal changes in E:A ratio, isovolumic relaxation time (IVRT), left ventricular end diastolic diameter (LVEDD), and cardiac output from week 0 to week 10. Differences between each treatment group vs. saline control are shown: * *p* < 0.05. Data shown on graphs are mean ± standard deviation.

**Figure 4:**
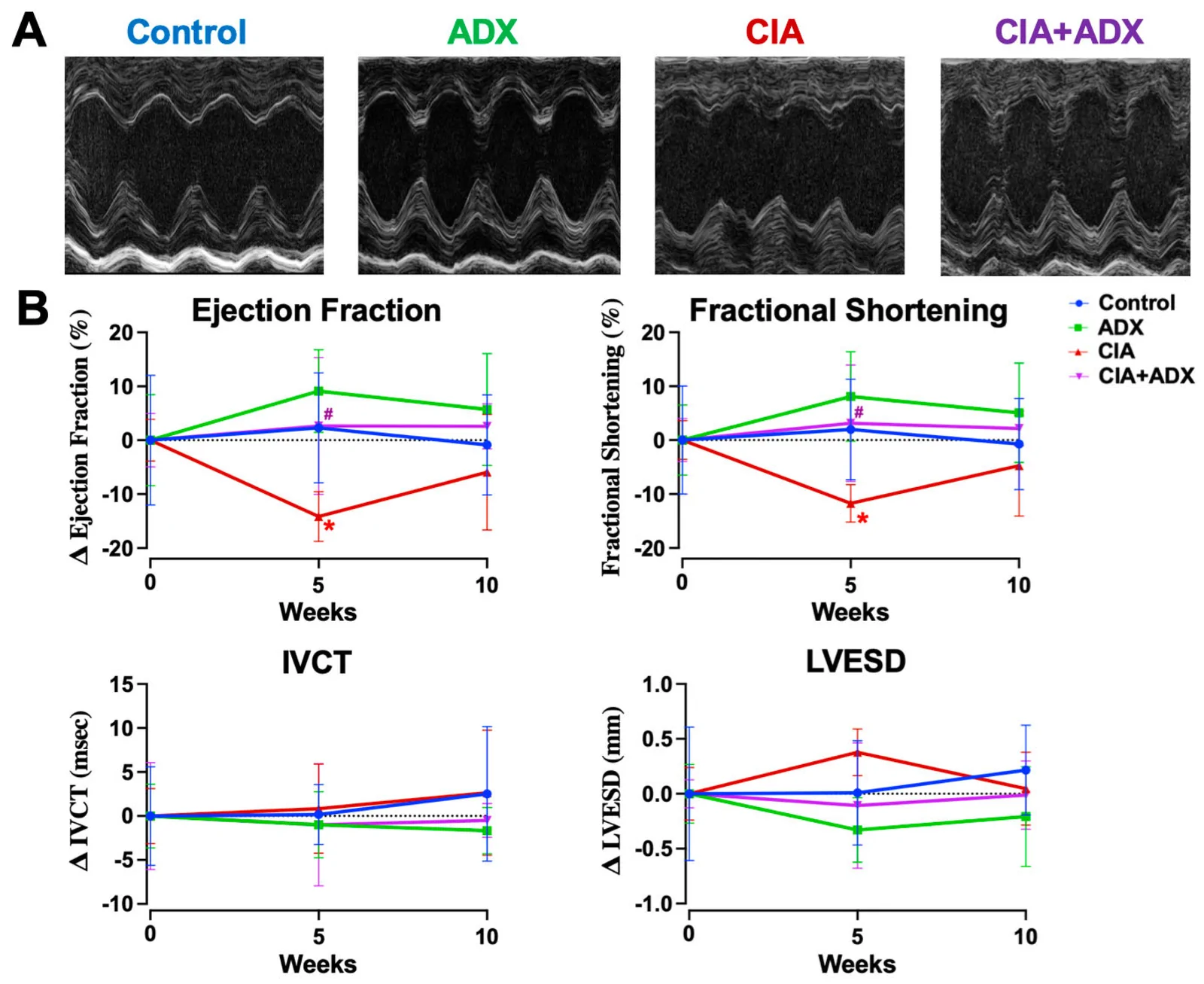
ADX-246 Attenuates Systolic Dysfunction in Mice with Early CIA. (**A**) Representative M-Mode echocardiograms from control (n = 5), ADX (n = 5), CIA (n = 6), and CIA + ADX (n = 5) mice at week 5. (**B**) Longitudinal changes in ejection fraction, isovolumic contraction time (IVCT), and left ventricular end systolic diameter (LVESD) from week 0 to week 10. Differences between each treatment group vs. saline control are shown: * *p* < 0.05. Differences between CIA + ADX and CIA group are shown: # *p* < 0.05. Data shown on graphs are mean ± standard deviation.

**Figure 5:**
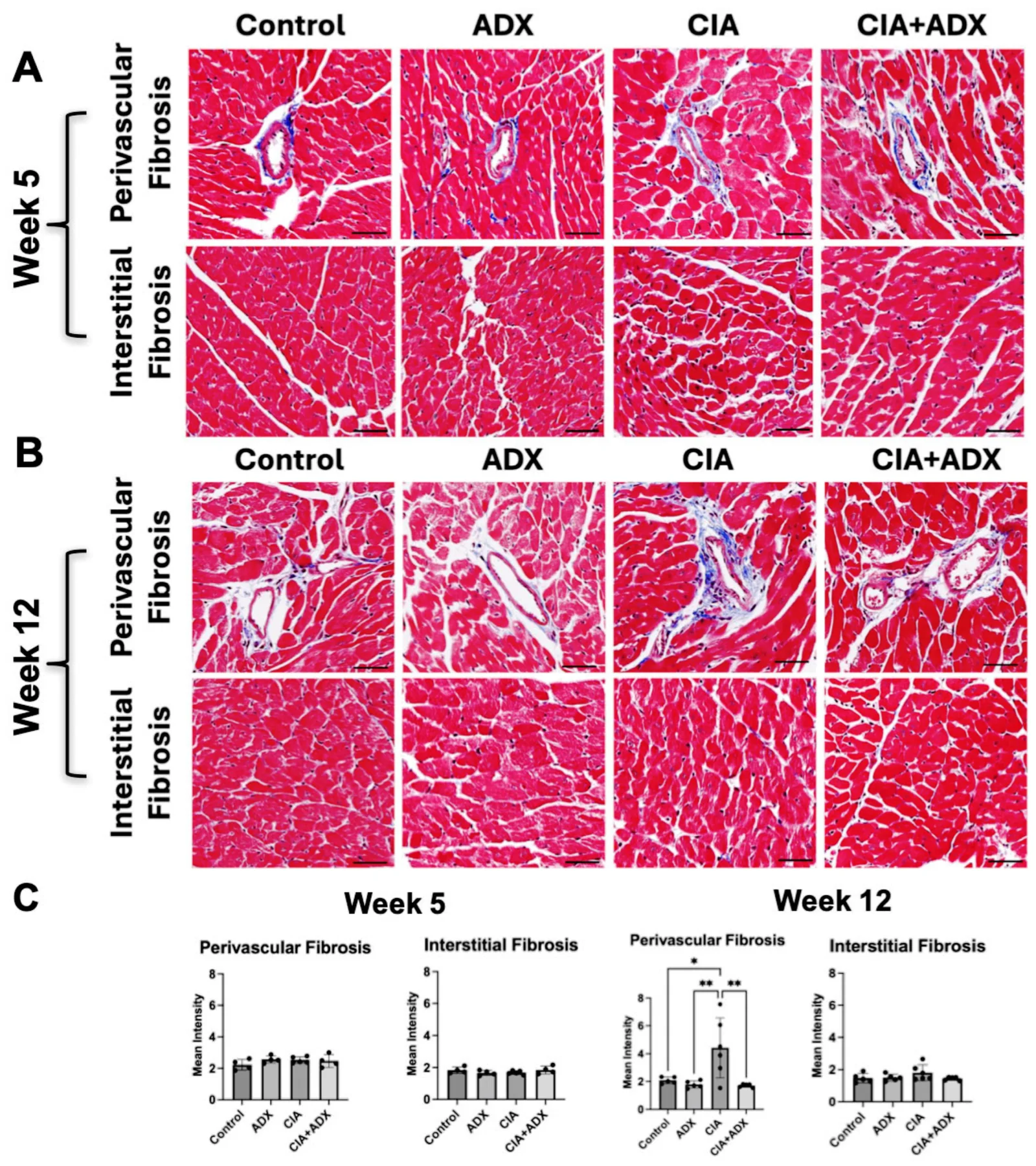
ADX-246 Attenuates Perivascular Fibrosis in Mice with Chronic CIA. Mice were sacrificed at (**A**) week 5 (control (n = 4), ADX (n = 4), CIA (n = 5), and CIA + ADX (n = 4)) and (**B**) week 12 (control (n = 5), ADX (n = 5), CIA (n = 6), and CIA + ADX (n = 5)), and left ventricular tissues were sectioned. Masson’s trichrome was performed and collagen deposition (blue) indicative of fibrosis was quantified. Representative 20× images are shown on panel (**A**,**B**). Scale bar at the bottom indicates 50 μm. Quantifications are shown on panel (**C**). Differences between groups are shown: * *p* < 0.05; ** *p* < 0.01. Data shown on graphs are mean ± standard deviation.

**Figure 6:**
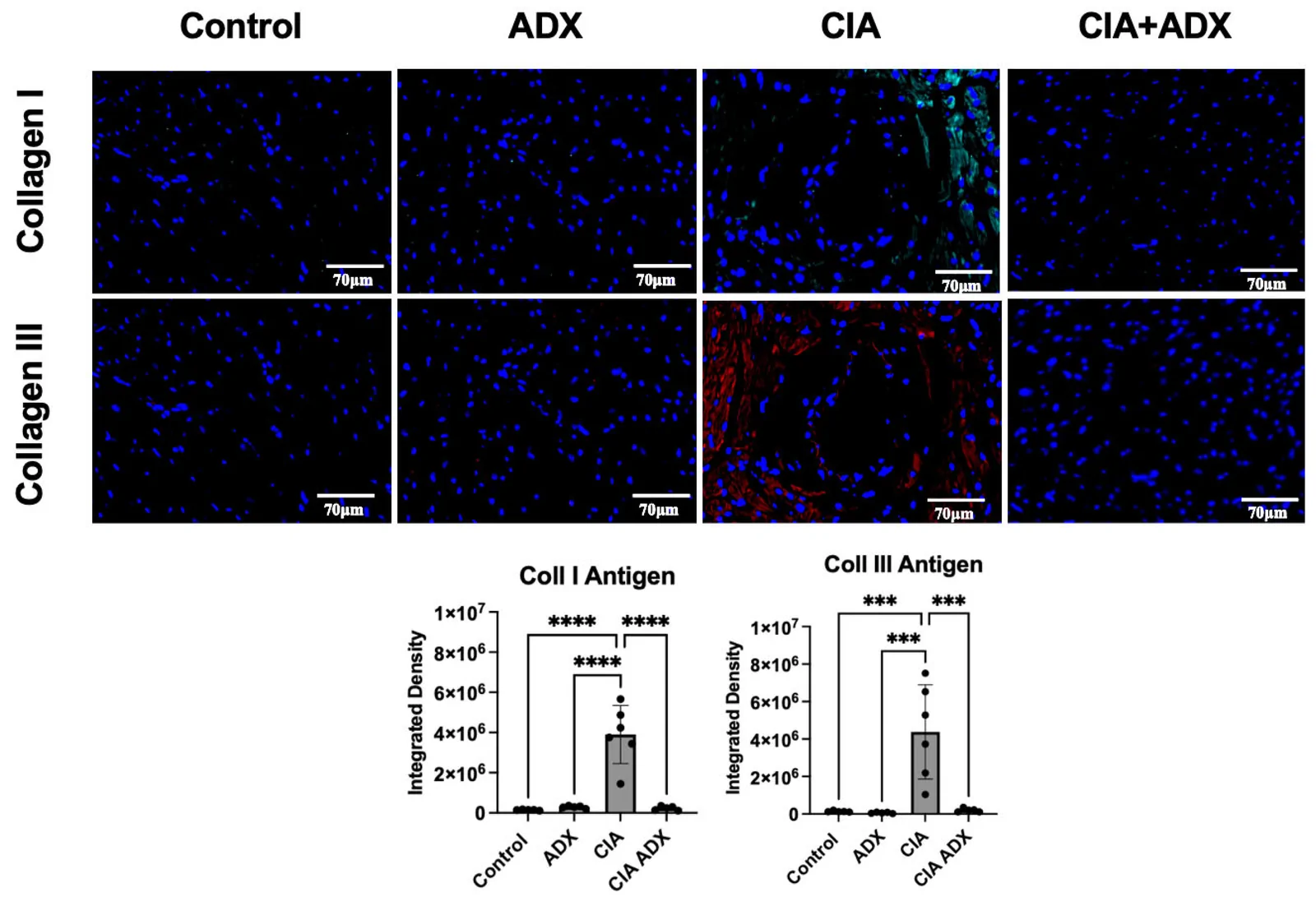
ADX-246 Attenuates Perivascular Collagen I and Collagen III Depositions in Mice with Chronic CIA. Immunohistochemistry was performed on left ventricular tissues to stain for collagen I (cyan), collagen III (red), and DAPI (blue). Representative 20× images around the perivascular regions are shown. Scale bar at the bottom indicates 70 μm. Intensities of collagen pixels were quantified in mice from each group: control (n = 4), ADX (n = 4), CIA (n = 5), and CIA + ADX (n = 4). Differences between groups are shown: *** *p* < 0.001; **** *p* < 0.0001. Data shown on graphs are mean ± standard deviation.

**Figure 7:**
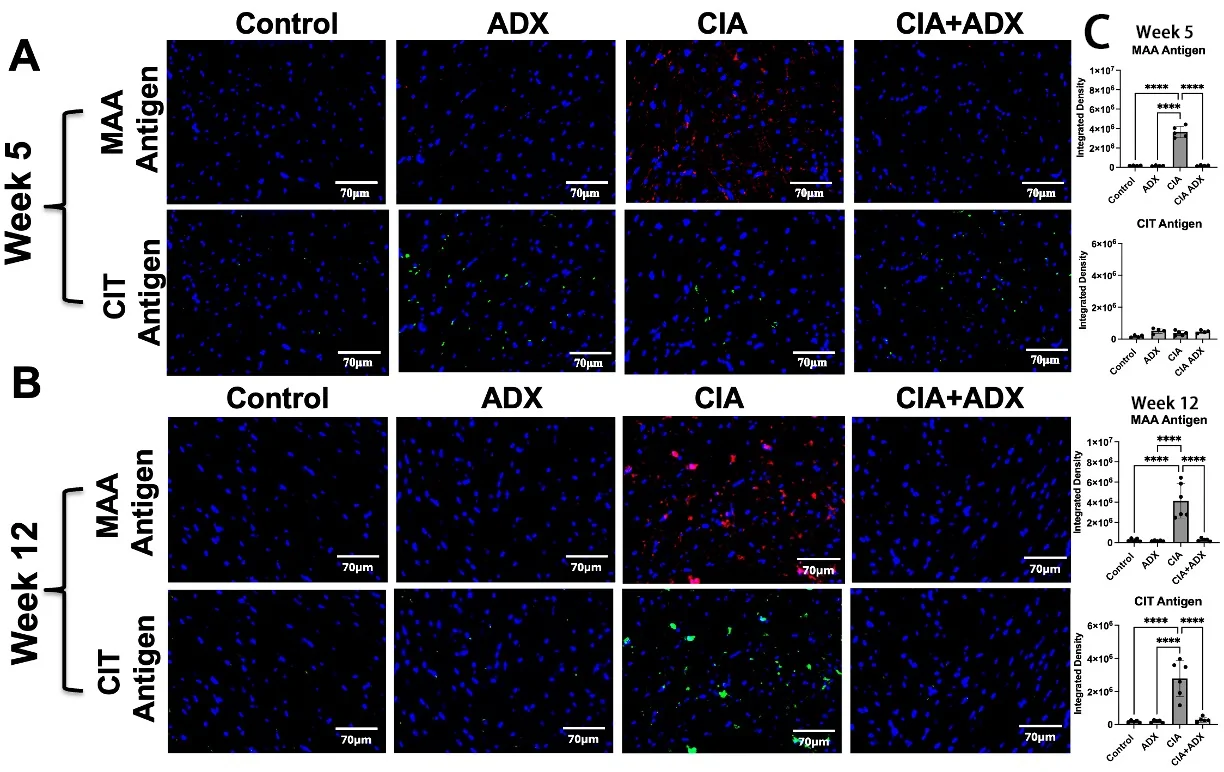
ADX-246 Prevents Myocardial Malondialdehyde Acetaldehyde (MAA) and Citrulline (CIT) Deposition in Mice with CIA. Mice were sacrificed at (**A**) week 5 (control (n = 4), ADX (n = 4), CIA (n = 5), and CIA + ADX (n = 4)) and (**B**) week 12 (control (n = 5), ADX (n = 5), CIA (n = 6), and CIA + ADX (n = 5)), and left ventricular tissues were sectioned. Immunohistochemistry was performed to stain for MAA (red), CIT (green), and DAPI (blue). Representative 20× images are shown on panel (**A**,**B**). Scale bar at the bottom indicates 70 μm. Quantifications are shown on panel (**C**). Moderate co-localization (r^2^ = 0.64) between MAA and CIT antigens was found in CIA mice at week 12. Differences between groups are shown: **** *p* < 0.0001. Data shown on graphs are mean ± standard deviation.

**Figure 8:**
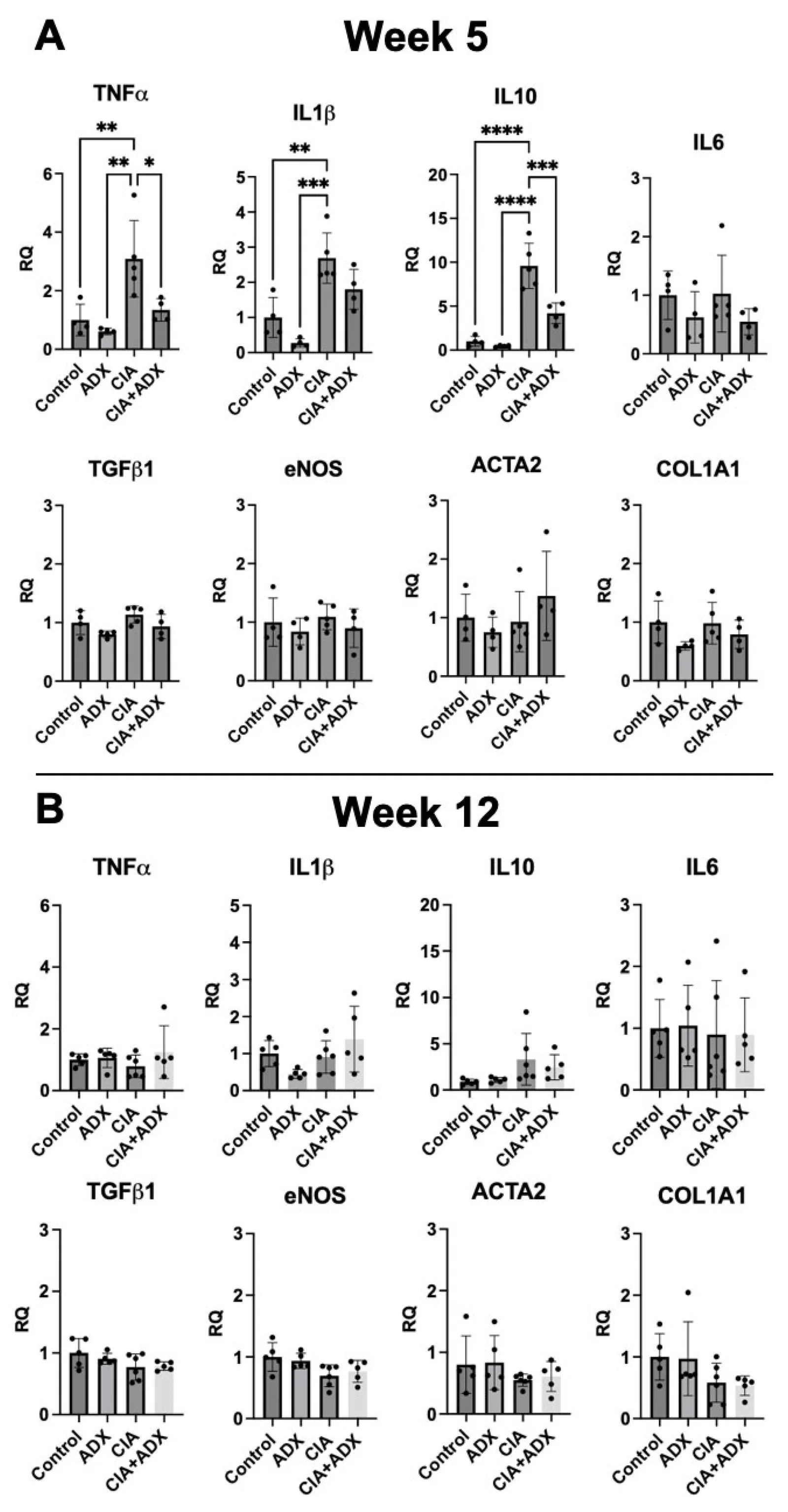
ADX Attenuated Pro-inflammatory Gene Expression in Left Ventricular Tissues of Mice with Early CIA. Mice were sacrificed at (**A**) week 5 (control (n = 4), ADX (n = 4), CIA (n = 5), and CIA + ADX (n = 4)) and (**B**) week 12 (control (n = 5), ADX (n = 5), CIA (n = 6), and CIA + ADX (n = 5)), and ventricular tissues were homogenized. RT-PCR was performed to measure gene expressions of *TNFα*, *IL1β*, *IL10*, *IL6*, *TGFβ1*, *eNOS*, *ACTA2*, and *COL1a1*. Relative quantification (RQ) compared to control is shown on *y*-axis. Differences between groups are shown: * *p* < 0.05; ** *p* < 0.01; *** *p* < 0.001; **** *p* < 0.0001. Data shown on graphs are mean ± standard deviation.

**Figure 9:**
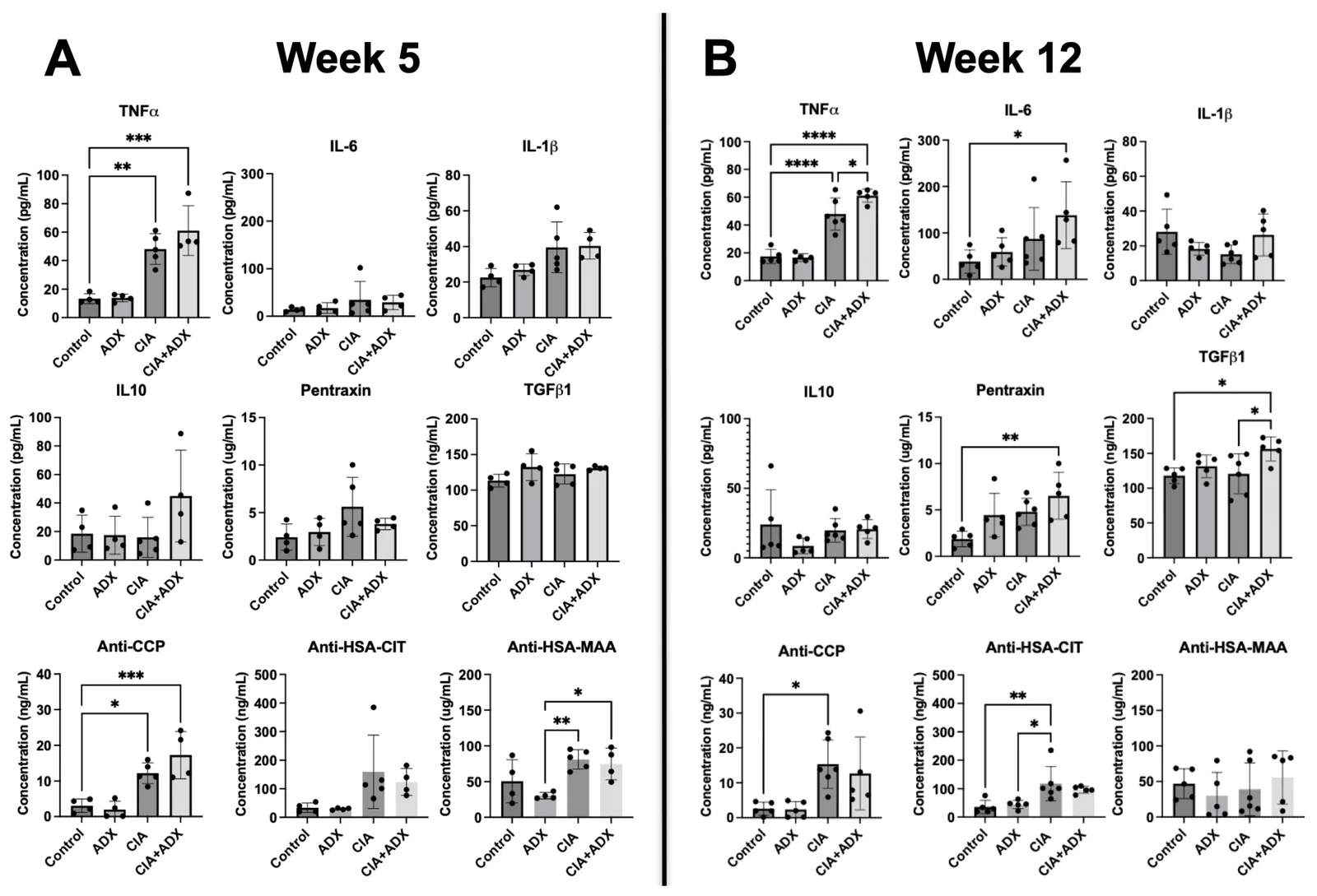
ADX-246 Did Not Attenuate Systemic Inflammation or Autoantibody Levels in Mice with CIA. Mice were sacrificed at (**A**) week 5 (control (n = 4), ADX (n = 4), CIA (n = 5), and CIA + ADX (n = 4)) and (**B**) week 12 (control (n = 5), ADX (n = 5), CIA (n = 6), and CIA + ADX (n = 5)). Serum inflammatory and fibrotic markers and autoantibody levels were quantified by ELISA. Differences between groups are shown: * *p* < 0.05; ** *p* < 0.01; *** *p* < 0.001; **** *p* < 0.0001. Data shown on graphs are mean ± standard deviation.

**Figure 10:**
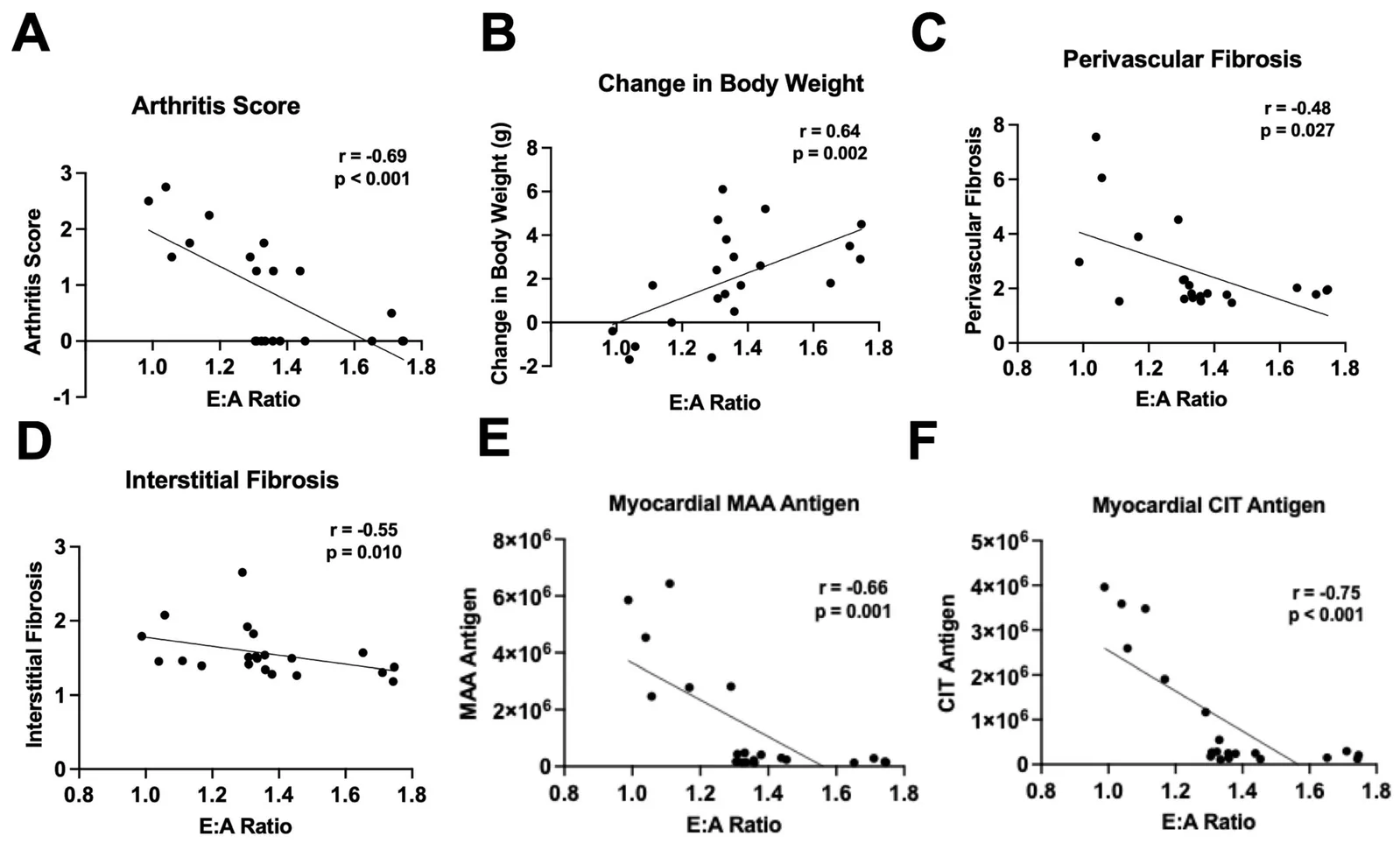
Correlations of Select Measurements with a Measure of Diastolic Dysfunction in Mice with Chronic CIA. Cross-sectional correlation between E:A ratio (at week 10 (control (n = 5), ADX (n = 5), CIA (n = 6), and CIA + ADX (n = 5))) and (**A**) arthritis score, (**B**) change in body weight, (**C**) left ventricular perivascular fibrosis, (**D**) left ventricular interstitial fibrosis, (**E**) myocardial MAA antigen levels, and (**F**) myocardial CIT antigen levels at the end point of the study (at week 12) from all four groups of mice (n = 21) are graphed. Nonparametric Spearman’s correlation coefficient (r) and *p* value are shown for each graph.

**Figure 11:**
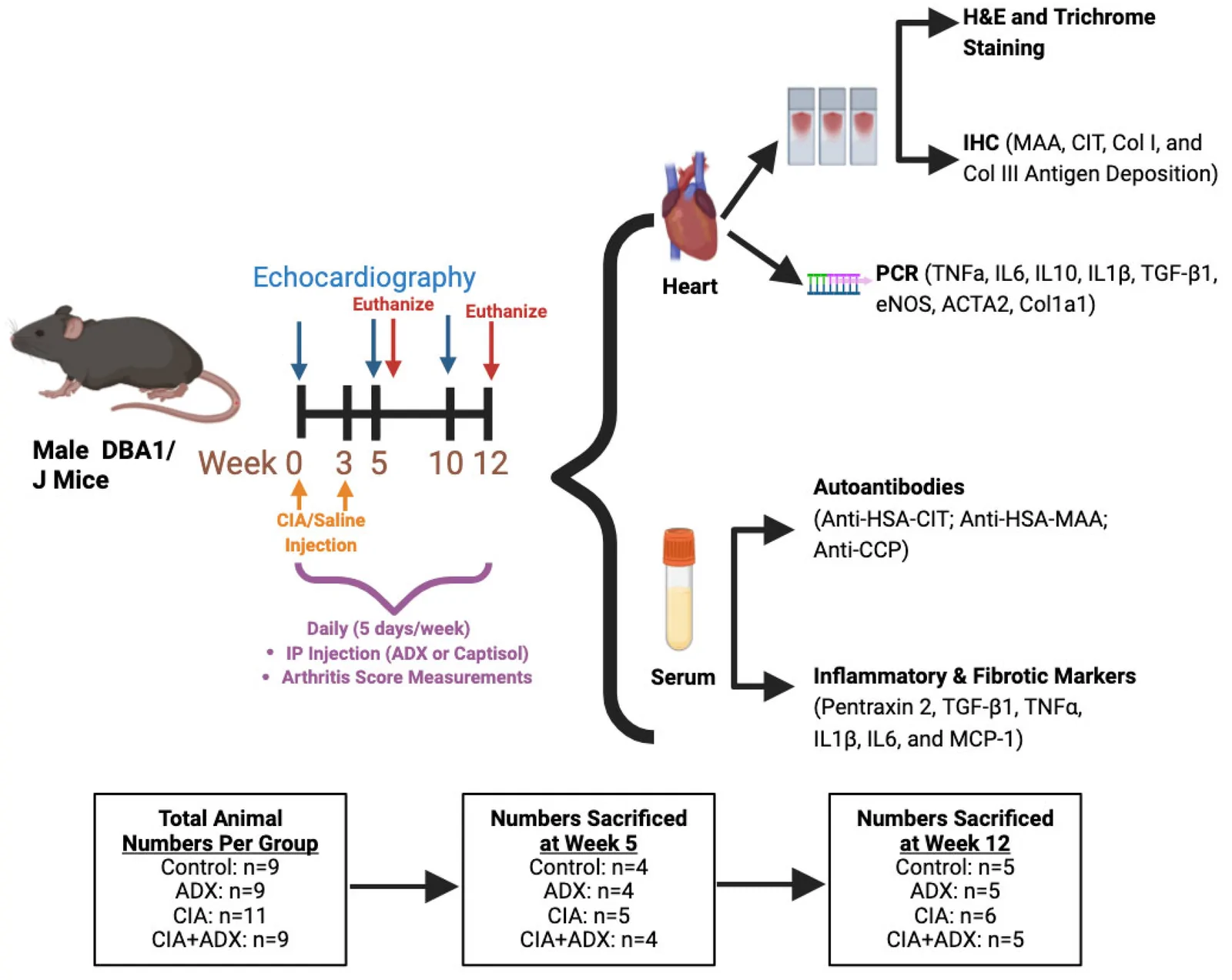
Animal Study Schema. A total of 38 male DBA/1J mice were randomly assigned to 4 groups (control n = 9; ADX-246 n = 9; CIA n = 11; CIA + ADX-246 n = 9). CIA was induced via standard protocol at weeks 0 and 3, with saline injection serving as negative control. Mice received daily (5 days/week) IP injection of either ADX-246 or Captisol as vehicle control. Transthoracic echocardiography was performed every 5 weeks. A total of 17 mice (control n = 4; ADX-246 n = 4; CIA n = 5; CIA + ADX-246 n = 4) were euthanized at week 5, and the remaining mice (control n = 5; ADX-246 n = 5; CIA n = 6; CIA + ADX-246 n = 5) were euthanized at week 12.

## Data Availability

The original contributions presented in this study are included in the article/Supplementary Materials. Further inquiries can be directed to the corresponding author.

## Supplementary Materials

The following supporting information can be downloaded at: https://www.mdpi.com/article/10.3390/ijms27198478/s1.

## Abbreviations

The following abbreviations are used in this manuscript:

ACPA	Anti-Citrullinated Protein Antibody
CCP	Cyclic Citrullinated Peptide
CIA	Collagen-Induced Arthritis
CIT	Citrullination
DAPI	4′,6-Diamidino-2-Phenylindole
EF	Ejection Fraction
FS	Fractional Shortening
HF	Heart Failure
HFpEF	Heart Failure with Preserved Ejection Fraction
HFrEF	Heart Failure with Reduced Ejection Fraction
HSA	Human Serum Albumin
HSA-CIT	Citrullinated Human Serum Albumin
HSA-MAA	Malondialdehyde–Acetaldehyde-Adducted Human Serum Albumin
IVCT	Isovolumic Contraction Time
IVRT	Isovolumic Relaxation Time
LVEDD	Left Ventricular End-Diastolic Diameter
LVESD	Left Ventricular End-Systolic Diameter
MAA	Malondialdehyde Acetaldehyde
PAD	Peptidylarginine Deiminase
PTM	Post-Translational Modification
RA	Rheumatoid Arthritis
RASP	Reactive Aldehyde Species
RQ	Relative Quantification
RU	Relative Unit
