# Supplementary material for: Attenuation of Cardiac Dysfunction in the Collagen-Induced Arthritis Mouse Model Mediated by a Reactive Aldehyde Species Inhibitor
Source: Int J Mol Sci. 2026 Sep 23;27(19):8478. doi: 10.3390/ijms27198478 (PMC13665015; doi:10.3390/ijms27198478)
Supplement: Supplementary file 1 [file ijms-27-08478-s001.zip › ijms-4559537-supplementary.pdf]

Attenuation of Cardiac Dysfunction in the Collagen Induced Arthritis Mouse Model Mediated by a Reactive Aldehyde Species Inhibitor

Wenxian Zhou[1], Hannah J. Johnson[1], Michael J. Duryee[1,2], Ali Namvaran[3], Aradhana Thapa[1], Cole Blasing[1], Amy Nelson[4], Engle E. Sharp[1], Carlos D. Hunter[1,2], Bryan Hackfort[5], Bryant R. England[1,2], Tate M. Johnson[1,2], Daniel R. Anderson[6], Adam Lazorchak[7], Robert G. Bennett[2,8,9], Jill A. Poole[4], Keshore Bidasee[3], Geoffrey M. Thiele[1,2] and Ted R. Mikuls[1,2,*]

[1]Department of Internal Medicine, Division of Rheumatology & Immunology, University of Nebraska Medical Center, Omaha, NE, USA
[2]Veterans Affairs Nebraska-Western Iowa Health Care System, Omaha, NE, USA
[3]Department of Pharmacology & Experimental Neuroscience, University of Nebraska Medical Center, Omaha, NE, USA
[4]Department of Internal Medicine, Division of Allergy and Immunology, University of Nebraska Medical Center, Omaha, NE, USA
[5]Department of Cellular and Integrative Physiology, University of Nebraska Medical Center, Omaha, NE, USA
[6]Department of Internal Medicine, Division of Cardiovascular Medicine, University of Nebraska Medical Center, Omaha, NE, USA
[7]Aldeyra Therapeutics, Lexington, MA, USA
[8]Department of Internal Medicine, Division of Diabetes, Endocrinology and Metabolism, University of Nebraska Medical Center, Omaha, NE, USA
[9]Department of Biochemistry and Molecular Biology, University of Nebraska Medical Center, Omaha, NE, USA
*Correspondence: Ted R. Mikuls, MD, MSPH; 986270 Nebraska Medicine, Omaha, NE, USA 68198-6270; email: tmikuls@unmc.edu; phone (402) 559-8168; fax (402) 559-6788

Supplemental Material

Figure S1
Table S1
Table S2
Table S3
ELISA Method
Major Resources Table S4

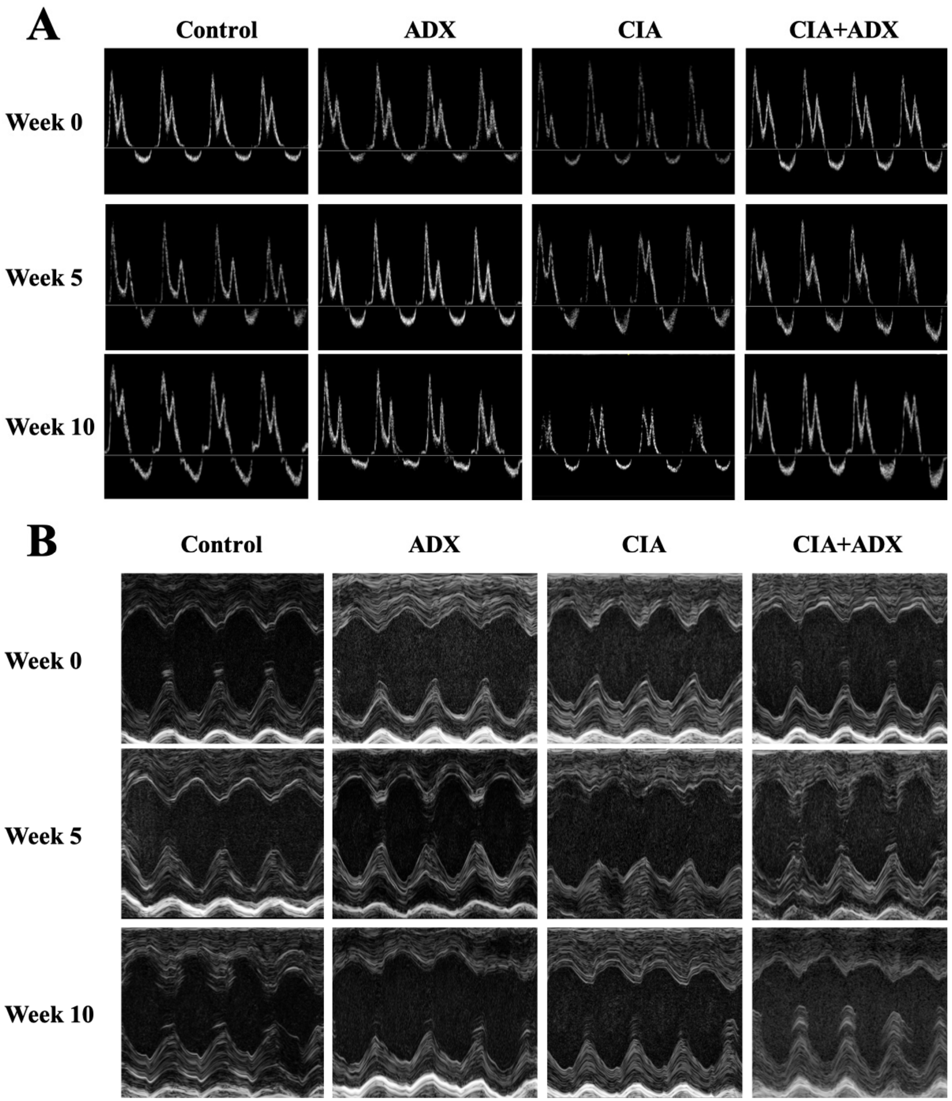

Figure S1. Mice Echocardiography. Representative **A)** pulsed-wave and **B)** M-mode echocardiogram from control (n=5), ADX (n=5), CIA (n=6), and CIA+ADX (n=5) mice at week 0, 5, and 10 are shown.

	Week 0				Week 5				Week 10			
	Control	ADX	CIA	CIA+ADX	Control	ADX	CIA	CIA+ADX	Control	ADX	CIA	CIA+ADX
**n**	5	5	6	5	5	5	6	5	5	5	6	5
**Body Weight (g)**	22.2 ± 0.9	21.0 ± 1.7	21.4 ± 1.0	21.6 ± 2.2	23.4 ± 1.2	22.6 ± 1.5	22.2 ± 1.4	22.5 ± 2.4	25.0 ± 1.1	24.5 ± 1.5	20.6 ± 1.6	22.6 ± 2.0
**Heart Rate (BPM)**	447 ± 49	443 ± 27	469 ± 34	458 ± 47	424 ± 90	470 ± 41	493 ± 36	431 ± 54	441 ± 42	484 ± 42	470 ± 14	447 ± 48
**Ejection Fraction (%)**	71.3 ± 12.1	68.5 ± 8.5	77.4 ± 3.9	68.2 ± 5.0	73.6 ± 10.2	77.7 ± 7.7	63.3 ± 4.6	70.9 ± 12.7	70.5 ± 9.3	74.2 ± 10.4	71.5 ± 10.7	70.8 ± 4.2
**Fractional Shortening (%)**	40.6 ± 10.0	37.9 ± 6.5	45.2 ± 3.6	37.4 ± 4.0	42.6 ± 9.3	46.0 ± 8.3	33.4 ± 3.5	40.6 ± 10.8	39.9 ± 8.4	42.9 ± 9.2	40.4 ± 9.4	39.6 ± 3.2
**Stroke Volume (µL)**	33.8 ± 5.1	33.8 ± 5.8	34.0 ± 7.0	36.5 ± 4.0	38.5 ± 7.3	36.8 ± 8.4	28.2 ± 7.9	36.2 ± 5.4	41.7 ± 4.0	34.7 ± 8.2	27.3 ± 6.2 **	40.2 ± 6.0 #
**Cardiac Output (mL/min)**	14.7 ± 4.0	15.0 ± 3.0	15.8 ± 2.8	16.7 ± 2.4	16.3 ± 3.8	17.4 ± 4.3	13.7 ± 3.2	15.6 ± 3.0	18.4 ± 2.4	16.8 ± 4.6	12.9 ± 3.2 *	17.8 ± 2.1
**IVCT (msec)**	14.2 ± 5.6	15.0 ± 3.6	16.8 ± 3.1	15.8 ± 6.1	14.3 ± 3.4	14.0 ± 3.7	17.6 ± 5.1	14.8 ± 6.9	16.7 ± 3.7	13.3 ± 2.6	19.4 ± 3.1	15.3 ± 1.9
**LVESD (mm)**	2.04 ± 0.61	2.15 ± 0.27	1.80 ± 0.24	2.23 ± 0.13	2.05 ± 0.48	1.82 ± 0.29	2.18 ± 0.21	2.13 ± 0.57	2.26 ± 0.41	1.94 ± 0.46	1.85 ± 0.33	2.22 ± 0.31
**MV E (mm/s)**	631 ± 87	630 ± 25	581 ± 32	646 ± 66	553 ± 70	606 ± 50	615 ± 73	576 ± 88	638 ± 75	706 ± 76	511 ± 54 *	621 ± 85
**MV A (mm/s)**	418 ± 49	437 ± 67	403 ± 47	440 ± 101	387 ± 87	421 ± 62	463 ± 85	398 ± 88	439 ± 75	504 ± 83	462 ± 36	441 ± 81
**E:A Ratio**	1.51 ± 0.18	1.46 ± 0.19	1.45 ± 0.13	1.51 ± 0.23	1.46 ± 0.21	1.46 ± 0.21	1.30 ± 0.15	1.48 ± 0.21	1.48 ± 0.21	1.42 ± 0.20	1.11 ± 0.11 *	1.43 ± 0.16 #
**LVRT (msec)**	15.3 ± 1.7	16.2 ± 1.3	16.8 ± 1.0	15.5 ± 3.8	14.8 ± 1.7	13.8 ± 1.4	17.6 ± 2.7	15.5 ± 1.6	14.5 ± 2.0	15.0 ± 3.5	21.4 ± 4.0 **	15.0 ± 1.8 #
**LVEDD (mm)**	3.39 ± 0.61	3.45 ± 0.19	3.28 ± 0.31	3.57 ± 0.08	3.55 ± 0.35	3.38 ± 0.31	3.28 ± 0.35	3.53 ± 0.38	3.74 ± 0.23	3.38 ± 0.38	3.10 ± 0.24 *	3.66 ± 0.32 #
**E:E' Ratio**	22.7 ± 1.5	24.2 ± 3.5	22.5 ± 4.4	25.8 ± 4.6	23.8 ± 4.9	18.6 ± 3.1	30.7 ± 5.1	22.0 ± 3.1 #	24.3 ± 6.5	21.2 ± 0.9	21.2 ± 4.6	23.2 ± 2.0

Table S1. Body Weight and Cardiac Parameters in Mice Over Time. Body weight and cardiac function of mice at week 0, 5, and 10 are shown. Data shown are mean ± standard deviation. Differences between each treatment group vs. saline control are shown: * p<0.05; ** p<0.01. Differences between CIA+ADX and CIA group are shown: # p<0.05.

	**IVRT**	**LVEDD**
**Arthritis Score**	r=0.61; p=0.003	r=-0.47; p=0.033
**Change in Body Weight**	r=-0.62; p=0.003	r=0.40; p=0.072
**Perivascular Fibrosis**	r=0.42; p=0.060	r=-0.27; p=0.24
**Interstitial Fibrosis**	r=0.31; p=0.176	r=-0.35; p=0.12
**Myocardial MAA Antigen**	r=0.54; p=0.011	r=-0.43; p=0.053
**Myocardial CIT Antigen**	r=0.60; p=0.004	r=-0.48; p=0.027

Table S2.Measurements that Significantly Correlates with Diastolic Dysfunction in Chronic CIA Mice. Correlation between indices of diastolic dysfunction (IVRT and LVEDD) at week 10 and arthritis score, change in body weight, left ventricular perivascular fibrosis, left ventricular interstitial fibrosis, myocardial MAA antigen, and myocardial CIT antigen at the end point of the study (at week 12) are calculated. Spearman's correlation coefficient (r) and p value are shown. Correlation with p<0.05 are highlighted.

ADX Compounds	Chemical Structure	Lot #	Purity (%w/w)	Associated Patent
ADX-203	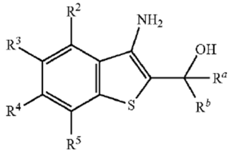	05BPN023	98.1	Brockman et al. 2024. HETEROCYCLIC ALDEHYDE TRAPPING COMPOUNDS AND USES THEREOF. U.S. Patent Application No. 18,568,566.
ADX-207	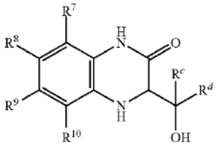	05NAG015	98.7	Brockman et al. 2024. HETEROCYCLIC ALDEHYDE TRAPPING COMPOUNDS AND USES THEREOF. U.S. Patent Application No. 18,568,566.
ADX-210	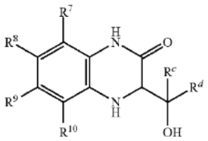	RLS-C-1084-34	99.9	Brockman et al. 2024. HETEROCYCLIC ALDEHYDE TRAPPING COMPOUNDS AND USES THEREOF. U.S. Patent Application No. 18,568,566.
ADX-246	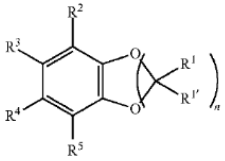	RLS-C-1161-54	99.3	Brockman et al. 2024. HETEROCYCLIC ALDEHYDE TRAPPING COMPOUNDS AND USES THEREOF. U.S. Patent Application No. 18,568,566.
ADX-248	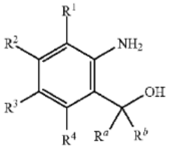	01BAL010	98.2	Brockman et al. 2024. HETEROCYCLIC ALDEHYDE TRAPPING COMPOUNDS AND USES THEREOF. U.S. Patent Application No. 18,568,566.
ADX-253	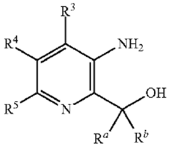	05NAG036	99.8	Brockman et al. 2024. HETEROCYCLIC ALDEHYDE TRAPPING COMPOUNDS AND USES THEREOF. U.S. Patent Application No. 18,568,566.

ADX-743	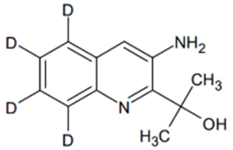	RLS-C-1262-42	99.8	Brady et al. 2017. TOXIC ALDEHYDE RELATED DISEASES AND TREATMENT. U.S. Patent No. 9,687,481.
ADX-629	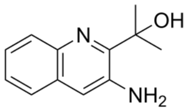	2491-1909-00553	100.0	Brady et al. 2017. TOXIC ALDEHYDE RELATED DISEASES AND TREATMENT. U.S. Patent No. 9,687,481.

Table S3. Chemical Structure, Purity, and Associated Patent for each ADX Compounds. The complete chemical structures of ADX-629 and ADX-743 and the core chemical structures of the remaining ADX compounds with proprietary substituents (R groups) masked are shown. In addition, lot number, purity, and associated patent for each of the ADX compounds listed in this paper are shown. The patents provide further details relating to the chemical synthesis and properties of the ADX compounds.

ELISA Method

Preparation of Modified Proteins

To evaluate the specificity of antibody responses to citrullinated or MAA-modified structures, human serum albumin (HSA) was modified as follows. HSA was citrullinated (HSA-CIT) using rabbit skeletal muscle peptidylarginine deiminase (PAD; Sigma) as previously described (PMID: 15899032). Citrullination of HSA was verified by ELISA using an anti-citrulline monoclonal antibody (PMID: 11870872). For MAA modification, 2 mg HSA was reacted with 2 mM MDA and 1.0 mM acetaldehyde (AA) in 0.1 M phosphate buffer (pH 7.2) containing 2 mM DTPA and 2 mM PA at 37 °C for 3 days, followed by dialysis against three changes of 0.1 M sodium phosphate buffer for 24 h at 4 °C (PMID: 11453728). MAA modification of HSA (HSA-MAA) was assessed by measuring characteristic MAA fluorescence (excitation 398 nm; emission 460 nm) using a Turner Biosystems LS-5B spectrofluorimeter (Sunnyvale, CA).

Anti-CIT and Anti-MAA ELISA

Serum antibodies directed against HSA-CIT and HSA-MAA were measured by ELISA. HSA-CIT and HSA-MAA were coated separately onto MaxiSorp ELISA plates (Thermo Fisher) at 2 µg/well and incubated overnight at 4 °C in 0.1 M bicarbonate buffer (pH 9.6). Plates were blocked with 2% casein buffer (pH 7.3 in PBST) for 1 h at room temperature. Mouse serum was diluted 1:1000 in 2% casein buffer and incubated for 2 h at room temperature. HRP-conjugated goat anti-mouse IgG (Jackson ImmunoResearch) was added at a 1:5000 dilution and incubated for 1 h at room temperature. Plates were developed with 1× 3,3′,5,5′-tetramethylbenzidine (TMB) substrate for 20 min at room temperature, and the reaction was stopped with 2 N sulfuric acid. Absorbance at 450 nm was measured using an Epoch plate reader (Agilent, Santa Clara, CA) and analyzed using Gen5 software (Agilent). Data are presented as relative units (RUs) of the specific antibody isotype detected.

Major Resources Table S4

Antibodies

Targeting Antigen	Vendor	Catalog #	Working Concentration	RRID
Mouse anti-CIT F95 IgMk	MilliporeSigma	MABN328	1:100	AB_2938608
A488 Donkey Anti-Mouse IgM	Jackson Immuno Research	715-545-140	1:1000	AB_2340845
Rabbit anti-MAA IgG	Generated and Validated in lab	NA	1:100	NA
Zenon 594 Rabbit IgG Labeling	Invitrogen	Z25307	1:1000	AB_2736956
Rabbit anti-Collagen I IgG Cy5	Bioss	bs-10423R-Cy5	1:100	NA
Rabbit anti-Collagen III IgG	Invitrogen	PA1-28870	1:100	AB_1956965
DAPI	Thermo Fisher	D1306	1:10,000	NA

Others

Name	Vendor	Catalog #
Human Serum Albumin	Akron Biotechnology LLC	NC1535793
Malondialdehyde	MilliporeSigma	63287
Acetaldehyde	MilliporeSigma	402788
Complete Freund's Adjuvant (5mg/mL)	Chondrex	7023
Incomplete Freund's Adjuvant	Chondrex	7024
Immunization Grade Chicken Type II Collagen	Chondrex	20011

ADX246	Aldeyra Therapeutics	ABP/1176/3A/12
Captisol	Aldeyra Therapeutics	RPCH9150
Isoflurane	Cardinal Health	803250
Allprotect Tissue Reagent	Qiagen	76405
RIPA Buffer	Thermo Fisher	89900
Protease Inhibitor Cocktail	MilliporeSigma	11697498001
Phenylmethylsulfonyl fluoride (PMSF)	MilliporeSigma	10837091001
BCA Assay	Thermo Fisher	23225
Precision Plus Protein Dual Color Standards	BIO-RAD	1610374
Casein	MilliporeSigma	70955
Rneasy Mini Kit	Qiagen	74104
High-Capacity RNA-to-cDNA Kit	Applied Biosystems	665544
TaqMan Gene Expression Mix	Applied Biosystems	4369016
Mouse TNF Expression Assays	Thermo Fisher	Mm00443258_m1
Mouse IL6 Expression Assays	Thermo Fisher	Mm00446190_m1
Mouse Il1b Expression Assays	Thermo Fisher	Mm00434228_m1
Mouse eNOS Expression Assays	Thermo Fisher	Mm00435217_m1
Mouse ACTA2 Expression Assays	Thermo Fisher	Mm01204962_gH
Mouse Tgfb1 Expression Assays	Thermo Fisher	Mm01178820_m1
Mouse I10 Expression Assays	Thermo Fisher	Mm00439616_m1
Mouse Col1a1 Expression Assays	Thermo Fisher	Mm00801666_g1
Mouse Pentraxin 2 ELISA Kit	R&D Systems	MPTX20
Anti-Cyclic CCP ELISA Kit	Thermo Fisher	FCCP600
Mouse TGF-β1 ELISA Kit	R&D Systems	DB100C
U-Plex Custom Mouse Biomarker Group 1	Meso Scale Discovery	K15069L-1
